# Learning the Orientation of a Loosely-Fixed Wearable IMU Relative to the Body Improves the Recognition Rate of Human Postures and Activities

**DOI:** 10.3390/s19132845

**Published:** 2019-06-26

**Authors:** Michael B. Del Rosario, Nigel H. Lovell, Stephen J. Redmond

**Affiliations:** 1 Graduate School of Biomedical Engineering, UNSW, Sydney, NSW 2052, Australia; 2 UCD School of Electrical and Electronic Engineering, University College Dublin, Belfield, 4 Dublin, Ireland; 3 UCD Centre for Biomedical Engineering, University College Dublin, Belfield, 4 Dublin, Ireland

**Keywords:** quaternion, smartphone, feature engineering, human activity recognition, sensor fusion

## Abstract

Features were developed which accounted for the changing orientation of the inertial measurement unit (IMU) relative to the body, and demonstrably improved the performance of models for human activity recognition (HAR). The method is proficient at separating periods of standing and sedentary activity (i.e., sitting and/or lying) using only one IMU, even if it is arbitrarily oriented or subsequently re-oriented relative to the body; since the body is upright during walking, learning the IMU orientation during walking provides a reference orientation against which sitting and/or lying can be inferred. Thus, the two activities can be identified (irrespective of the cohort) by analyzing the magnitude of the angle of shortest rotation which would be required to bring the upright direction into coincidence with the average orientation from the most recent 2.5 s of IMU data. Models for HAR were trained using data obtained from a cohort of 37 older adults (83.9 ± 3.4 years) or 20 younger adults (21.9 ± 1.7 years). Test data were generated from the training data by virtually re-orienting the IMU so that it is representative of carrying the phone in five different orientations (relative to the thigh). The overall performance of the model for HAR was consistent whether the model was trained with the data from the younger cohort, and tested with the data from the older cohort after it had been virtually re-oriented (Cohen’s Kappa 95% confidence interval [0.782, 0.793]; total class sensitivity 95% confidence interval [84.9%, 85.6%]), or the reciprocal scenario in which the model was trained with the data from the older cohort, and tested with the data from the younger cohort after it had been virtually re-oriented (Cohen’s Kappa 95% confidence interval [0.765, 0.784]; total class sensitivity 95% confidence interval [82.3%, 83.7%]).

## 1. Introduction

Wearable movement sensors, i.e., sensors that incorporate inertial measurement units (IMUs) and barometric altimeters, have been championed as tools that will positively impact health care [1]. These technologies have demonstrated their utility in the remote monitoring of patient rehabilitation [2], as well as the clinical analysis of gait [3], from which parameters can be extracted to predict falls in the elderly [4,5]. They have come to prominence in the management of Parkinson’s disease [6], objectively quantifying patient tremor [7], and tracking the impact of the disease on their gait [8]. Moreover, wearables have been adopted for the longitudinal monitoring of physical activity, which can be used to identify those at risk of developing type-2 diabetes [9] and obesity [10].

### 1.1. Multiple Sensors or a Single Sensor?

The number of sensors that an individual needs to wear for adequate human activity recognition (HAR) is dependent on three factors: (i) the number of activities to be recognized, (ii) the location of the sensor(s) on the body, and (iii) the nature of the sensors (i.e., some arbitrary combination of accelerometers, gyroscopes, barometric pressure sensors, magnetometers, etc.). If the activities to be recognized involve the movement of each of the body’s limbs (e.g., lunges, push ups, hand stands, etc.), multiple sensors may need to be worn on the body at specific locations to obtain measurements that allow the movement to be accurately identified. Wearing a single sensor on the body is sufficient if the model for HAR is only identifying gross body movements (e.g., standing, sitting, walking, running), placing the sensor near the body’s center of mass is ideal (i.e., the thigh) [11].

In either case, wearing sensors at different locations on the body will increase the performance of a model for HAR [12], but at the expense of user compliance and burden [13], particularly if the sensor(s) must be placed somewhere uncomfortable or unsightly [14]. Consequently, wearable sensor systems for population-based studies are predominantly of the single-sensor variety [15,16], ideally integrating seamlessly into the daily lives of users (e.g., embedded within a watch, necklace, sock or belt) [17].

### 1.2. Smartphone-Based Human Activity Recognition

The dramatic recent increase in smartphone ownership [18,19] coupled with society’s dependence on smartphones [20,21] has changed the paradigm. If individuals carry their device with them, the measurements from the smartphone’s IMU and barometric altimeter can be analyzed to identify the users’ gross body movements throughout the day. As a result, smartphones can be used as tools for the purposes of physical rehabilitation, weight loss, etc., in which the ability to recognize human activity is essential [22]. Finally, the greater penetration of smartphones amongst those of a lower socioeconomic status [23] would enable population-based interventions to be conducted at a reduced cost and with a wider reach. There are different models for HAR which can be adopted [24].

#### 1.2.1. Fixed-to-the-Body

In this scenario, the IMUs embedded within smartphones are used in place of dedicated IMU devices to: (i) detect falls [25], (ii) monitor activities of daily living [26], (iii) monitor the performance of soccer and field hockey athletes [27], and (iv) swimmers [28]. These models assume that the smartphone will be worn at one location on the body and that the device’s orientation relative to the body segment on which it is worn is known *a priori* and does not change during the monitoring period because it is held in place with a strap or similar apparatus.

#### 1.2.2. Body-Position-Dependent

Conversely, models can be designed under the assumption that the smartphone is not strapped to the body and will be placed in either the user’s pants/chest pocket [29], or hand or bag [30]. These models do not require the user’s smartphone habits to change (as with those in Section 1.2.1) to accommodate the device being fixed to the body, however, this makes it challenging to infer the user’s postural orientation due to the variability with which the sensor can be oriented in the pocket with respect to the body (i.e., the initial orientation of the device relative to the body segment on which it is worn cannot be controlled, and the orientation of the device can vary over time since it is not firmly fixed to the body).

#### 1.2.3. Body-Position-Dependent

The final variant relaxes all constraints with respect to the smartphone’s position and orientation on the body. Models are robust to device transitions from hand to pants/chest pocket [31], or bag, at any moment [32,33]. A trade-off for this robustness (compared to those discussed in Section 1.2.1 or Section 1.2.2) is that it can be difficult to determine body posture due to variability in both the device’s location and its relative orientation to the body.

### 1.3. Extracting Information for Activity Recognition

There are two broad supervised learning approaches which have emerged to process sensor measurements for HAR: (i) feature engineering and classification, or (ii) deep learning.

#### 1.3.1. Accounting for Variability in Device Orientation and Position

A number of pre-processing techniques have been proposed to reduce the variability in sensor measurements due to the inconsistency of the location and/or orientation with which the device is placed on the body. Khan et al. demonstrated that linear discriminant analysis (LDA) can be used to improve a classification algorithm’s ability to distinguish between transitions from sitting to standing (and vice versa), and standing to lying (and vice versa) [34]. They also illustrated how kernel discriminant analysis (KDA) can estimate both the interclass and intraclass variance of features used to separate periods of walking, running, walking upstairs, and walking downstairs [35]. Henpraserttae et al. applied eigenvalue decomposition to tri-axial accelerometer measurements to infer the device’s orientation with respect to the body by assuming that most of the acceleration due to body movement is in the forward direction, and that the vertical axis can be inferred from the low-pass filtered acceleration [36].

Yurtman and Barshan proposed another transformation based on singular value decomposition (SVD). They first pre-processed the data from a tri-axial accelerometer, tri-axial gyroscope, and tri-axial magnetometer so that it had unit variance, before SVD was applied to the entire time sequence to make the sensor measurements agnostic to the device’s orientation [37]. Yurman et al. followed their seminal work with another method which combined the measurements from the accelerometer, gyroscope, and magnetometer, to estimate the sensor’s orientation within the global frame of reference when it is firmly fixed to the body. The differential quaternions they generated, which estimated the relative change in the device’s orientation between time intervals, enabled the raw sensor measurements to be expressed in a reference frame invariant to the sensor’s orientation [38].

#### 1.3.2. Feature Engineering and Classification

Feature engineering involves the application of domain knowledge to design hand-crafted features [39,40] which describe the changing characteristics of the data with respect to time. These features and labels (i.e., the human activities to be recognized which are temporally aligned with the feature values) are input to a classification algorithm (e.g., decision tree, support vector machine, Naïve Bayes, artificial neural networks, etc.) which tries to derive the best mathematical model that separates the labeled observations, based on the statistic distributions of those features.

#### 1.3.3. Deep Learning/Deep Neural Networks

Alternatively, domain knowledge can be replaced with a standalone artificial intelligence solution that abstracts the entire feature extraction and classification process. Deep learning approaches are a natural extension to artificial neural networks, comprised of numerous neurons and layers, which attempt to learn both the Best Features and model for HAR by using the training labels to determine the value of the neurons’ weights at each layer in the network [41]. The performance of these approaches are dependent on the network’s architecture and the quality and quantity of the training data. While convolutional neural networks [42], short-time Fourier transforms combined with temporal convolution layers [43], long/short term memory (LSTM) networks [44], or a combination of convolution, recurrent, and LSTM network layers [45], have all been shown to perform exceptionally well, they incur a considerable energy cost when running on a smartphone due to the demands of real-time processing [46].

### 1.4. Contribution

This paper addresses the limitations associated with methods for inferring postural orientation that are dependent on the sensor’s precise anatomical placement [47,48] by presenting a novel method (i.e., a hand-crafted feature) for identifying sedentary periods of activity that is robust to variability in the sensor’s orientation. The sensor’s orientation during walking periods is learned on-line and used as a reference for the upright body orientation (represented by the quaternion $q_{\mathrm{upright}}^{}$). Comparing the sensor’s recent average orientation (over a sliding window) to $q_{\mathrm{upright}}^{}$ enables standing and sedentary periods to be distinguished, regardless of the IMU’s orientation. It is important to distinguish between standing and sedentary activities due to their differing energy expenditure profiles [49,50]. Furthermore, there are definitive relationships between total sedentary time per day and: type-2 diabetes [51,52]; cardiovascular mortality; all-cause mortality [53,54]; and even cancer [55].

## 2. Materials

Wearable sensor data from our previous work [56], in which a cohort of twenty younger adults (15 male and five female) of ages 21.9 ± 1.7 years (Human Research Ethics Advisory, reference number 08/2012/48) and 37 older adults (25 male and 12 female) of age 83.9 ± 3.4 years (Human Research Ethics Committee, reference number HC12316) performed nine human activities whilst a smartphone was placed in their pants pocket, was used to evaluate the method proposed herein. The younger adults were able-bodied university students recruited from the University of New South Wales, Sydney, Australia. The older adults were recruited from a cohort of participants enrolled in an existing study on memory and aging at Neuroscience Research Australia (NeuRA), Sydney, Australia. These participants were community-dwelling and retirement village residents living in inner and eastern Sydney; aged 65+ years; English-speaking; with a mini-mental state examination (MMSE) score of 24 or above; no acute psychiatric condition with psychosis or unstable medical condition; not currently participating in a fall prevention trial.

Sensor data from the IMU and barometric altimeter were originally sampled at $f_{IMU}=100$ Hz and $f_{bar}=16$ Hz, respectively. The measurements from the IMU and altimeter were also re-sampled at 40 Hz and 20 Hz, respectively, to demonstrate the method’s ability to be adapted for a reduced sampling rate, thereby reducing the prospective power consumption of the algorithm. This is important because the usability of wearable sensors increases if they can operate continuously throughout the waking day [24,57].

Periods of human activity, originally labeled as elevator up/down, were relabeled as standing to focus on the clinical relevance of the activity rather than the wider context of the person being in an elevator; this naturally increased the classification performance by reducing the range of activities being classified. Additionally, sitting and lying were collectively re-labeled as sedentary. Consequently, the nine activities described in [56] were reduced to six: sedentary, standing, walking, walking upstairs (WU), walking downstairs (WD) and postural transitions (PT).

## 3. Methods

Note in the sections that follow, (i) quaternion multiplication (⊗) and conjugation (${}^{*}$) are defined in [58]; (ii) vectors are bold-faced (i.e., b); (iii) quaternions are bold-faced, italicized and normalized unless explicitly stated (i.e., q = $q\;/\;\left|\left|q\right|\right|$); (iv) vectors expressed in the sensor frame, or estimated in the global frame of reference, will be denoted with the superscripts ${}^{s}b$, and ${}^{g}b$, respectively; (v) a function will be denoted as *f*(…) with arguments inside the brackets.

### 3.1. Generating Data Representative of Different Orientations

**Figure 1:**
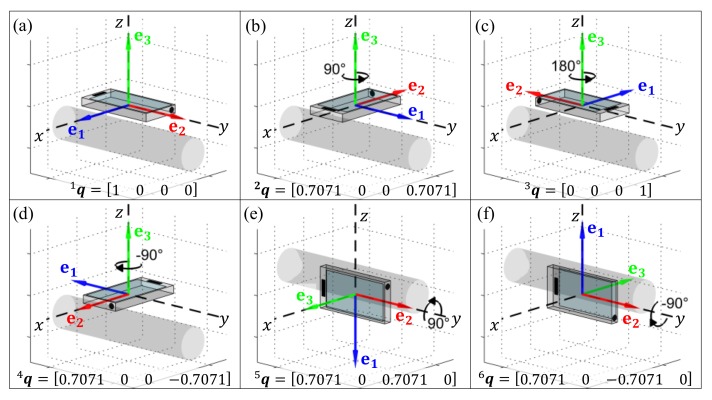
Six common ways that the inertial measurement units (IMU) might be placed in the pants pocket (assuming a seated position). The cylinder in each panel represents the orientation of the participant’s right thigh whilst seated (with the knee to the right-hand side of each image). The dashed lines labeled *x*, *y*, and *z* denote the original device reference frame, ${}^{1}q$, whilst the orthogonal basis defined by the vectors, $e_{1}^{}$, $e_{2}^{}$, and $e_{3}^{}$ illustrate the device orientation generated. In panels (**a**–**d**) the IMU is located on the anterior surface of the thigh (i.e., a pocket on the front of the pants), whilst in panels (**e**,**f**) the IMU is located on the lateral surface of the thigh (i.e., a pocket on the outer side of the pants).

Each quaternion in Figure 1a–f was used to transform the accelerometer and gyroscope data ($r_{\mathrm{acc}}$ and $r_{\mathrm{gyr}}$, respectively) collected in our previous work [56], into new accelerometer and gyroscope data ($v_{\mathrm{acc}}$ and $v_{\mathrm{gyr}}$, respectively) that would have been obtained if the smartphone were re-oriented in the pants pocket (Equation (1)). Note: (i) {$r_{\mathrm{acc}}$, $r_{\mathrm{gyr}}$, $v_{\mathrm{acc}}$, $v_{\mathrm{gyr}}$} ∈$R^{3}$; (ii) data from the barometric altimeter were not transformed as these scalar measurements are orientation invariant.

(1) $$\left[\begin{array}{cccc} 0 & v_{x}^{} & v_{y}^{} & v_{z}^{} \end{array}\right]=q⊗\left[\begin{array}{cccc} 0 & r_{x}^{} & r_{y}^{} & r_{z}^{} \end{array}\right]⊗q^{*}.$$

### 3.2. Estimating the Orientation of the IMU

The data generated in Section 3.1 were fused using the adaptive error-state Kalman filter (AESKF) for orientation estimation, developed in our previous work [59], to estimate the device’s orientation. The tuning parameters of the AESKF algorithm are listed in Table 1. Note, whilst there are many algorithms that can be used to estimate the IMU’s orientation, the AESKF was chosen for its computational efficiency relative to other algorithms [59].

#### 3.2.1. Removing the Heading from the Estimated Orientation

**Figure 2:**
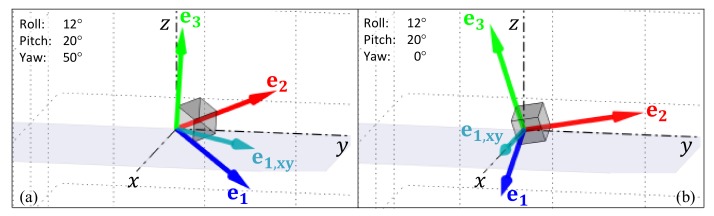
Effect of removing the yaw from an arbitrary orientation (the orthogonal basis defined by the vectors $e_{1}^{}$ (in blue), $e_{2}^{}$ (in red), $e_{3}^{}$ (in green) by aligning it with the x-axis of the standard basis x,y,z: (**a**) orientation, with an arbitrary yaw angle; (**b**) the same orientation with the yaw component removed (see Equations (2)–(5)). Note: the light blue vector is $e_{1,xy}^{}$, the $e_{1}^{}$ basis vector of the orientation projected to the xy plane; the pitch and roll angles are preserved.

The estimated orientation, $q_{\mathrm{AESKF},k}^{}$, had an arbitrary heading that did not contain any information about the orientation of the IMU on the individual’s body, since the person can face in any direction and perform the same activity. Consequently, this was removed by aligning the orientation, $q_{\mathrm{AESKF},k}^{}$, with the north-facing component of the standard basis, $e_{x}^{}$ = $\left[\begin{array}{ccc} 1 & 0 & 0 \end{array}\right]$. First, the x basis vector of the quaternion, $q_{\mathrm{AESKF},k}^{}$, was identified and projected to the xy-plane (Equation (2)). Once $x_{xy,k}^{}$ is determined, the quaternion, $q_{\mathrm{north},k}^{}$, that rotates the device orientation, $q_{\mathrm{AESKF},k}^{}$, northward can be calculated (Equations (4)–(6)). The resultant quaternion, $q_{k}^{}$, had a fixed heading, i.e., the yaw angle, ψ = 0 (see Figure 2b), which ensured that the shortest rotation between two quaternions (Section 4.2.2), did not contain any information about changes in the device’s heading which normally occur due to turning the body.

(2) $$x_{xy,k}^{}={\left[\begin{array}{ccc} q_{0}^{2}\;+\;q_{1}^{2}-q_{2}^{2}-q_{3}^{2} & 2(q_{1}^{}q_{2}^{}\;+\;q_{0}^{}q_{3}^{}) & 0 \end{array}\right]}_{k}^{}$$

(3) $$\theta_{k}^{}=\cos^{-1}\left(MM\left(x_{xy,k}^{}\cdot e_{x}^{}\right)\;/\;∥\begin{array}{c} x_{xy,k}^{} \end{array}∥\right)$$

(4) $$n_{k}^{}=x_{xy,k}^{}\times e_{x}^{}$$

(5) $$q_{\mathrm{north},k}^{}={\left[\begin{array}{cc} \cos(\frac{\theta }{2}) & n\cdot \sin(\frac{\theta }{2}) \end{array}\right]}_{k}$$

(6) $$q_{k}^{}=q_{\mathrm{north},k}^{}⊗q_{\mathrm{AESKF},k}^{}$$

#### 3.2.2. Smoothing the Estimated Orientation

The effects of the IMU shifting/re-orienting within the individual’s pants pocket as they move through the world were minimized by time-averaging the quaternion, $q_{k}^{}$, using a computationally-efficient one-pass method [60], to create a moving average (window size *N*) of the device orientation (Equation (7)) from the last 2.5 worth of data, ${\bar{q}}_{k}^{}$.

(7) $${\bar{q}}_{k}^{}=f_{q,\mathrm{avg}}^{}\left(q_{k-N+1}^{},\cdots ,q_{k}^{}\right),$$

see Appendix A Equation (A1).

## 4. Feature Extraction

The features in Table 2 were aggregated (using a sliding window with 50% overlap) using sensor data from the most recent 2.5 s. Features (1)–(4) were obtained by processing the sensor measurements with finite impulse response (FIR) linear phase filters, as described in our previous work [56]. Whilst novel features (5)–(8) are described in Section 4.1–Section 4.3.

### 4.1. Squared Magnitude of Pitch/Roll Angular Velocity

In our previous work [56], the three orthogonal gyroscope measurements were each band-pass filtered (between 1 and 20 Hz) to isolate the frequency components predominantly due to walking [61]. The squared magnitude of these three band-pass filtered signals at each time sample, $\omega_{\mathrm{bpf},k}^{2}$, was used to distinguish between active/inactive periods of activity. Alternatively, the measurements can be expressed in the estimated global frame of reference (GFR) using the device orientation, $q_{k}^{}$. The squared magnitude of the pitch/roll rotation, ${}^{g}\omega_{xy,k}^{2}$, can be isolated using Equations (8) and (9) since rotations about the vertical axis are primarily due to turning.

(8) $$\begin{array}{c} \begin{array}{c} {\left[\begin{array}{cccc} 0 & {}^{g}\omega_{x}^{} & {}^{g}\omega_{y}^{} & {}^{g}\omega_{z}^{} \end{array}\right]}_{k}=q_{k}^{}⊗{\left[\begin{array}{cccc} 0 & {}^{s}\omega_{x}^{} & {}^{s}\omega_{y}^{} & {}^{s}\omega_{z}^{} \end{array}\right]}_{k}⊗{\left(q_{k}^{}\right)}^{*} \end{array} \end{array}$$

(9) $$\begin{array}{c} \begin{array}{c} {}^{g}\omega_{xy,k}^{2}{=}^{g}\omega_{x,k}^{2}{+}^{g}\omega_{y,k}^{2} \end{array} \end{array}$$

### 4.2. Detecting Sedentary Periods

**Figure 3:**
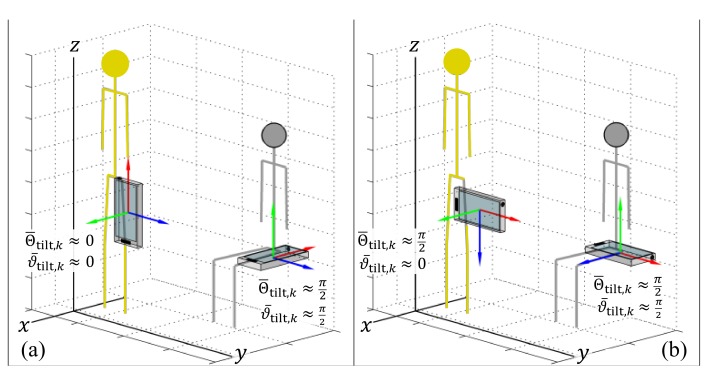
Two alternative methods for measuring the tilt. The limitations of the traditional tilt angle variable (i.e., the angle between the red basis vector and the *z*-axis of the global frame of reference (GFR)) are clear, making it impossible to separate standing (stick figure in gold) and sedentary periods (stick figure in grey). When the red basis vector: (**a**) runs along the length of the individual’s leg, the magnitude of the tilt angle changes by ≈$\frac{\pi }{2}$ radians (i.e., 90°) between standing and sedentary periods, making it easy to discriminate these postures; (**b**) red basis vector runs along the mediolateral axis of the individuals’s leg, so the magnitude of the tilt angle of the red vector remains relatively unchanged between standing and sedentary periods, resulting in confusion between the two postures.

The tilt angle, $\Theta_{\mathrm{tilt},k}^{}$, was previously used [56] to identify the postural orientation of the body relative to the global frame of reference (GFR) [47,48]. In this previous formulation, the magnitude of $\Theta_{\mathrm{tilt},k}^{}$ is dependent on one of the axes of the IMU (the *y*-axis in Figure 3a) remaining in coincidence with the long axis of the thigh. This constraint is apparent when the IMU is oriented such that another axis is aligned with the long axis of the thigh (Figure 3b), but is also a problem if the orientation of the IMU shifts in the pocket. A new approach is proposed in Section 4.2.2 which compares the average recent orientation with the average orientation during walking periods (i.e., the sensor’s orientation during walking periods is continuously learned and used to define the ‘upright’ orientation, against which all other orientations are compared).

#### 4.2.1. Estimate the Upright Orientation using the Orientation during Walking Periods

Walking periods were identified using the method proposed by Jiménez et al. [62] which analyzed the squared magnitude of the raw tri-axial gyroscope signal, ${}^{s}\omega_{k}^{2}$ (see Equation (10)), and the magnitude of the unbiased sample variance, $\varsigma_{\mathrm{acc},k}^{2}$ (see Equation (12)), of the squared magnitude of the raw tri-axial accelerometer signal, ${}^{s}a_{k}^{2}$ (see Equation (11)). When both signals are greater than pre-determined thresholds, the individual carrying the IMU was presumed to be walking (see Equation (13)). Note, *j* = k−N+1 in Equation (12), and $\varsigma_{\mathrm{acc},k}^{2}$ was calculated using a computationally-efficient method [60] with a sliding window of 0.25 s; i.e., *N* = ⌊0.25 ×$f_{\mathrm{IMU}}^{}\rfloor$.

(10) $${}^{s}\omega_{k}^{2}{=}^{s}{\left[\omega_{x}^{2}+\omega_{y}^{2}+\omega_{z}^{2}\right]}_{k}$$

(11) $${}^{s}a_{k}^{2}{=}^{s}{\left[a_{x}^{2}+a_{y}^{2}+a_{z}^{2}\right]}_{k}$$

(12) $$\varsigma_{\mathrm{acc},k}^{2}=\frac{1}{N-1}\left[\left(\sum_{i=j}^{k}{{(}^{s}a_{i}^{2})}^{2}\right)-\frac{1}{N}{\left({\sum_{i=j}^{k}}^{s}a_{i}^{2}\right)}^{2}\right]$$

(13) $$b_{\mathrm{walk},k}=\left\{\begin{array}{cc} 1, & \left(\omega_{k}^{2}>5\;\mathrm{rad}/s\right)\;\cap \;\left(\varsigma_{\mathrm{acc},k}^{2}>10\;m^{2}/s^{4}\right)\\ 0, & \mathrm{otherwise} \end{array}\right.$$

The scalar and vector components of each orientation, $q_{k}^{}$, which correspond to these walking periods are stored and used to calculate the ‘upright’ orientation, $q_{\mathrm{upright},k}^{}$ (see Equation (14) and Appendix A). Note: ${}^{⋆}$ denotes the set of *N* indices corresponding to the most recent 2.5 s of data for which $b_{\mathrm{walk},k}^{}$ = 1, and need not be a contiguous set of sample indices.

(14) $$q_{\mathrm{upright},k}^{}=f_{q,\mathrm{avg}}^{}\left(q_{{\left(k-N+1\right)}_{}^{⋆}}^{},\cdots ,q_{k_{}^{⋆}}^{}\right),$$

see Appendix A Equation (A1).

The approach presented herein improves upon the method proposed by Elvira et al. because: (i) an orientation algorithm is utilized [63] whose estimated inclination angle is immune to magnetic interference [64,65]; (ii) an adaptive error-state Kalman filter (AESKF) is used which enables the estimated orientation to be quickly corrected during ‘quasi-static’ periods [59]; (iii) the estimated heading (i.e., the yaw component, ψ°) is removed from the orientation estimated (the importance of which is demonstrated in Section 3.2.1); (iv) most importantly this method is believed to be the first to demonstrate the utility of the shortest rotation between two quaternions as a feature for HAR.

#### 4.2.2. Calculate the Shortest Rotation between the Upright Orientation and the Average Recent Orientation

Once the average recent orientation, ${\bar{q}}_{k}^{}$, and the upright orientation, $q_{\mathrm{upright},k}^{}$, are known, the magnitude of the shortest rotation between them (see Equation (15)) can be calculated (see derivation in Appendix B) and used to distinguish standing and sedentary (seated/lying) periods, regardless of the IMU’s orientation relative to the thigh.

(15) $$\vartheta_{\mathrm{tilt},k}^{}=f_{\mathrm{angle}}^{}\left(q_{\mathrm{upright},k}^{},{\bar{q}}_{k}^{}\right),$$

see Appendix B Equation (A2).

### 4.3. Estimating Velocity in the Vertical Direction of the GFR

The inertial acceleration in the sensor frame was obtained by measuring the magnitude of the Earth’s gravitational acceleration, $\left|\left|y_{a,0}\right|\right|$, (i.e., the accelerometer measurement during a quasi-static period, where the accelerometer is not moving) and expressing this measurement in the sensor frame of reference as ${}^{s}z_{k}^{}$ using the accelerometer-corrected attitude, $q_{k}$ (see Equations (16) and (17)). The acceleration due to gravity, as measured in the sensor frame of reference, ${}^{s}g_{\mathrm{ref},k}$, can then be subtracted from the raw accelerometer measurement, ${}^{s}y_{a,k}^{}$ (Equation (18)), to obtain the inertial acceleration in the sensor frame, ${}^{s}d_{a,k}$.

This acceleration can be expressed in the estimated GFR, ${}^{g}d_{a,k}^{}$ = ${\left[\begin{array}{ccc} {}^{g}d_{a,x}^{} & {}^{g}d_{a,y}^{} & {}^{g}d_{a,z}^{} \end{array}\right]}_{k}^{}$, using Equation (19). At this point, the sensor’s velocity in the vertical direction of the GFR can be estimated by fusing the vertical component of the acceleration, ${}^{g}d_{a,z,k}^{}$, with the barometric pressure sensor measurements, $p_{k}^{}$, using a complementary filter [66] or Kalman filter [67]. Assuming the external acceleration, ${\ddot{z}}_{k}^{}$ = ${}^{g}d_{a,z,k}^{}$, remains constant over the sampling interval, *T* = $\frac{1}{f_{\mathrm{IMU}}^{}}$, and the bandwidth of ${\ddot{z}}_{k}^{}$ is less than $\frac{f_{\mathrm{IMU}}^{}}{2}$, the time-propagation of the altitude, *z* and velocity, $\dot{z}$, can be modeled [68] according to Equation (20):

(16) $${}^{s}z_{k}^{}={\left[\begin{array}{ccc} 2\left(q_{1}q_{3}-q_{0}\right)q_{2}) & 2(q_{2}q_{3}+q_{0}q_{1}) & 2{\left(q_{0}\right)}^{2}-1+2{\left(q_{3}\right)}^{2} \end{array}\right]}_{k}^{}$$

(17) $${}^{s}{\overset{˘}{g}}_{\mathrm{ref},k}=\left|\left|{}^{s}y_{a,0}\right|\right|\cdot {}^{s}z_{k}^{}$$

(18) $${}^{s}d_{a,k}^{}{=}^{s}y_{a,k}{-}^{s}{\overset{˘}{g}}_{\mathrm{ref},k}$$

(19) $${\left[\begin{array}{cccc} 0 & {}^{g}d_{a,x}^{} & {}^{g}d_{a,y}^{} & {}^{g}d_{a,z}^{} \end{array}\right]}_{k}=q_{}^{}⊗{\left[\begin{array}{cccc} 0 & {}^{s}d_{a,x}^{} & {}^{s}d_{a,y}^{} & {}^{s}d_{a,z}^{} \end{array}\right]}_{k}^{}⊗q_{}^{*}$$

(20) $$\begin{array}{llllll} {\left[\begin{array}{c} z\\ \dot{z} \end{array}\right]}_{k}^{} & = & {\left[\begin{array}{cc} 1 & T\\ 0 & 1 \end{array}\right]}_{k}^{} & {\left[\begin{array}{c} z\\ \dot{z} \end{array}\right]}_{k-1} & +{\left[\begin{array}{c} \frac{T^{2}}{2}\\ T \end{array}\right]}_{k} & {\ddot{z}}_{k}\\ x_{k} & = & A_{k} & x_{k-1} & + & G_{k}\;u_{k}\;+w_{k} \end{array}$$

#### 4.3.1. Process Model

Imperfections in Equation (20), i.e., acceleration not being constant during the sampling interval, and noise in the acceleration input to the system, $u_{k}^{}$, prevent the system’s true state, x, from being observed. Consequently, the system’s state can only be estimated as ${\overset{˘}{x}}_{k}$, by combining the process model with measurements obtained directly from the system. The ‘prediction step’ (i.e., Equations (22) and (23)) produces an *a priori* estimate of the system’s state, ${\overset{˘}{x}}_{k}^{-}$, and covariance, $P_{k}^{-}$. Note: (i) $Q_{k}^{}$ is the process noise covariance matrix, (ii) $\frac{a_{m}^{}}{2}$≤$\sigma_{\mathrm{acc}}$≤$a_{m}^{}$, where $a_{m}^{}$ is the magnitude of the maximum acceleration the system will experience [68].

(21) $${\overset{˘}{x}}_{k}^{-}=A_{k}^{}{\overset{˘}{x}}_{k-1}^{+}+G_{k}^{}u_{k}^{}$$

(22) $$P_{k}^{-}=A_{k}^{}P_{k-1}^{+}A_{k}^{T}+Q_{k}^{}$$

(23) $$Q_{k}^{}=G_{k}^{}G_{k}^{T}\sigma_{\mathrm{acc}}^{2}=\left[\begin{array}{cc} \frac{1}{4}T^{4} & \frac{1}{2}T^{3}\\ \frac{1}{2}T^{3} & T^{2} \end{array}\right]\sigma_{\mathrm{acc}}^{2}$$

#### 4.3.2. Observation Model

The observation model (Equation (24)) transforms the state estimate, ${\overset{˘}{x}}_{k}^{}$, to the domain of the barometric pressure sensor $p_{k}^{}$ (i.e., it converts altitude (in m) to air pressure (hPa) [69]), and enables the measurement residual, ${\tilde{y}}_{k}$, to be calculated (Equation (26)). The measurement residual has a covariance $S_{k}^{}$ that combines the covariance of the *a priori* state estimate, $P_{k}^{-}$, and variance in the measurement from the barometric pressure sensor, $R_{k}^{}=\sigma_{\mathrm{bar}}^{2}$ (Equation (27)); i.e., the variance in the barometric pressure when the device remains stationary. The gain in the filter, $K_{k}$, can be determined by consolidating the covariances of the *a priori* state estimate and measurement residual (Equation (28)), thereby enabling the a posteriori state estimate, ${\overset{˘}{x}}_{k}^{+}$, and covariance, $P_{k}^{+}$, to be determined as described in Equations (29) and (30). Note: (i) $H_{k}^{}$ is the Jacobian of $h(x_{k}^{})$, that is, derivatives with respect to the elements of the state vector $x_{k}^{}$, evaluated at the estimate $x_{k}^{}={\overset{˘}{x}}_{k}^{}$; (ii) $I_{2}^{}$ a 2×2 identity matrix.

(24) $$h\left(x_{k}^{}\right)=p_{0}^{}{\left(1-\frac{z_{k}^{}}{44330.77}\right)}^{5.26}$$

(25) Hk=∂h∂z∂h∂z˙|h(x=xk)

(26) $${\tilde{y}}_{k}=p_{k}^{}-h\left({\overset{˘}{x}}_{k}^{-}\right)$$

(27) $$S_{k}^{}=H_{k}^{}P_{k}^{-}H_{k}^{T}+R_{k}^{}$$

(28) $$K_{k}^{}=P_{k}^{-}H_{k}^{T}S_{k}^{-1}$$

(29) $${\overset{˘}{x}}_{k}^{+}={\overset{˘}{x}}_{k}^{-}+K_{k}^{}{\tilde{y}}_{k}^{}$$

(30) $$P_{k}^{+}=\left(I_{2}^{}-K_{k}^{}H_{k}^{}\right)P_{k}^{-}$$

It is hoped that the Kalman-filtered velocity estimate, ${\dot{z}}_{k}$, was able to distinguish between walking periods, upstairs (${\dot{z}}_{k}>>0$), downstairs (${\dot{z}}_{k}<<0$), and on a level surface (${\dot{z}}_{k}\approx 0$). This would extend the utility of the Kalman-filtered velocity estimate, beyond applications in fall detection [70], for example.

## 5. Hierarchical Description of Human Activity

**Figure 4:**
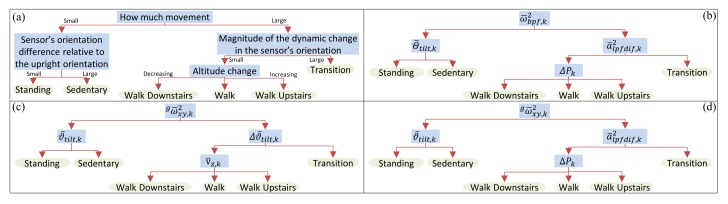
Illustration of how the activity classes can be separated using (**a**) a hierarchical description of human activity. A schematic for achieving the separation using the following features: (**b**) only original (i.e., features (1)–(4)), (**c**) only new (i.e., features (5)–(8)), and (**d**) best features from all eight old and New Features (i.e., features (3)–(6)) in Table 2. Each blue rectangle represents a classification and regression tree (CART) [73] implemented in MATLAB 2013b with ‘ClassificationTree.fit’. The CART algorithm used ‘uniform’ prior class probabilities to ensure that the thresholds selected accounted for any class imbalances.

**Figure 5:**
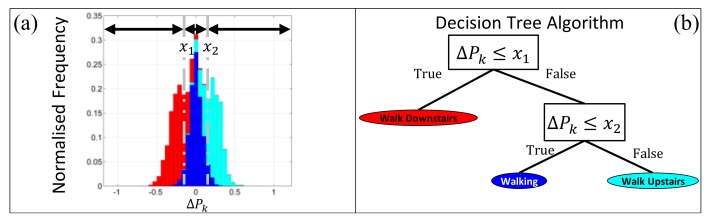
(**a**) Normalized frequency histogram for the $\Delta P_{k}$ feature and three activity classes (walk downstairs (in red), walking (in blue), and walk upstairs (in light blue), visualized as stacked bars); (**b**) An example of the decision tree, used at each node of the HMHA; i.e., the blue rectangles in Figure 4. $x_{1}^{}$ and $x_{2}^{}$, are derived from Figure 5a according to the classification and regression tree [73].

Rather than use one supervised machine learning algorithm to perform HAR, a hierarchical model of human activity (HMHA) [71,72] was devised and translated into a feature-based model (see Figure 4). A decision tree based on the classification and regression tree (CART) algorithm developed by Brieman [73] was trained for each node of the model and pruned so that there is only one leaf node for each activity class (see an example in Figure 5b). This approach minimized over-fitting [74], ensured that the model was easily interpreted, and makes the process of HAR tractable in the event of misclassification [75]. In addition, the weights of each class were balanced when the decision tree was trained to ensure that the thresholds selected accounted for any class imbalances [76].

## 6. Models and Performance Metrics

### 6.1. Performance at a High Sampling Rate

A number of models were developed in which a model for recognizing human activity was trained using all of the sensor data collected from the younger and/or older cohorts using either: (i) the Original Features, i.e., features (1)–(4) in Table 2; (ii) the New Features (i.e., features (5)–(8) in Table 2), and; (iii) four pairs of features (i.e., features 1 and 5; features 2 and 6; features 3 and 7; features 4 and 8 from Table 2) were provided to four separate instances of the CART algorithm to select the four Best Features to separate the human activities into distinct classes according to the structure of the HMHA described in Figure 4a; these pairings represent features which are similar in terms of the information they captures, e.g., features 1 and 5 capture angular velocity information in subtly different ways.

The robustness of each model for HAR was evaluated by virtually re-orienting the device (as described in Section 3.1) to obtain data from the younger and/or older cohorts that are representative of five different device orientations (see Figure 1b–f). Each model’s performance was evaluated by training the model with either of the younger and/or older cohorts data and testing the model with either of the younger and/or older cohorts data after it had been virtually re-oriented, using 10-fold cross-validation. Ninety-five percent confidence intervals (95%CIs) were calculated for the: Cohen’s kappa (κ) and total classification sensitivity (%), as well as the sensitivity (%) and specificity (%) of each activity class. This process is repeated for the ‘Best Features’ (determined in the above search procedure).

### 6.2. Translating Performance to Different Sampling Rates

Finally, the HMHA are evaluated by training the model with data from the younger and/or older cohort at either (i) the original sampling rate (i.e.,the IMU sampled at 100 Hz, and the barometric altimeter data sampled at 16 Hz), or (ii) a reduced sampling rate (i.e., the IMU resampled at 40 Hz, and the barometric altimeter data resampled at 20 Hz), and testing the model with the virtually rotated data from the younger and/or older cohort at the reciprocal sampling rate to determine if the performance and thresholds of the model are consistent. The metrics reported in Section 6.1 were also used to evaluate the model’s performance.

## 7. Results and Discussion

### 7.1. Comparing Features Using Shannon Entropy

**Figure 6:**
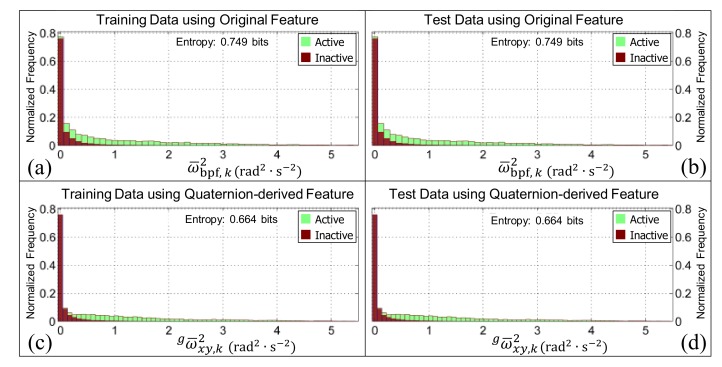
The normalized histograms of the active (i.e., the walking, walking upstairs, walking downstairs, and postural transition classes pooled together) and inactive (i.e., the standing and sedentary classes pooled together) classes for the features ${\bar{\omega }}_{\mathrm{bpf},k}^{2}$ and ${}^{g}{\bar{\omega }}_{xy,k}^{2}$. Panels (**a**,**c**) are generated from the training data (i.e., the pooled data from the younger and older cohorts, respectively). Panels (**b**,**d**) are generated from the test data (i.e., the pooled data from the younger and older cohort after they have been virtually re-oriented using the quaternions in Figure 1b–f). The bar charts in all panels are ‘stacked’. Note that the Shannon entropy for the quaternion-derived feature is both smaller and consistent, irrespective of the data it is calculated from, which suggests that it will be better at distinguishing the activity classes and is orientation invariant.

When the Shannon entropy [77] of the training datasets (i.e., Figure 6a,c) or testing datasets (i.e., Figure 6b,d) were compared for the features ${}^{g}{\bar{\omega }}_{xy,k}^{2}$ and ${\bar{\omega }}_{\mathrm{bpf},k}^{2}$, two things become evident. Firstly, both features appear to be orientation invariant because the Shannon entropy is constant whether or not it is calculated from the training data or test data (i.e., here the test data was the virtually re-oriented training data). Secondly, the Shannon entropy was reduced by 0.085 bits when the quaternion-derived feature was used in place of the original feature proposed in our previous work, showing an improvement in the separation of the class distributions [56].

**Figure 7:**
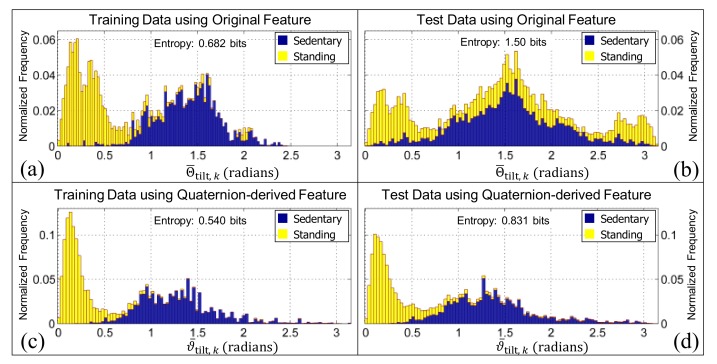
The normalized histograms of the sedentary and standing classes (illustrated in Figure 4) for the features ${\bar{\Theta }}_{\mathrm{tilt},k}$ and ${\bar{\vartheta }}_{\mathrm{tilt},k}$. Panels (**a**,**c**) are the histograms obtained when the training data are used (i.e., the pooled data from the younger and older cohorts, respectively). Panels (**b**,**d**) are the histograms obtained when the test data are used (i.e., the pooled data from the younger and older cohort after it had been virtually re-oriented using each of the quaternions in Figure 1b–f). The bar charts in all panels are ‘stacked’. Note that the entropy for the quaternion-derived feature is consistently smaller, which suggests that it will be better at distinguishing between activity classes.

The features ${\bar{\Theta }}_{\mathrm{tilt},k}$ and ${\bar{\vartheta }}_{\mathrm{tilt},k}$ can be used to further separate the inactive class into standing and sedentary (i.e., sitting or lying) classes. When the Shannon entropy was calculated for both tilt angle features using the training data (see Figure 7a,c, respectively) the Shannon entropy [77] dropped by 0.142 bits when ${\bar{\vartheta }}_{\mathrm{tilt},k}$ was used in place of ${\bar{\Theta }}_{\mathrm{tilt},k}$. A more pronounced difference of 0.669 bits was observed between ${\bar{\Theta }}_{\mathrm{tilt},k}$ and ${\bar{\vartheta }}_{\mathrm{tilt},k}$ when the Shannon entropy was calculated using the test data (see Figure 7b,d, respectively). Whilst the Shannon entropy of ${\bar{\vartheta }}_{\mathrm{tilt},k}$ increases by 0.291 bits when the test data are used in place of the training data, the shape of the normalized frequency distribution is more consistent for all device re-orientations when compared with ${\bar{\Theta }}_{\mathrm{tilt},k}$ which increased by 0.818 bits for the re-oriented (test) data. This suggests that the quaternion-derived feature, ${\bar{\vartheta }}_{\mathrm{tilt},k}$ is more robust to how a smartphone is initially placed in the pocket.

**Figure 8:**
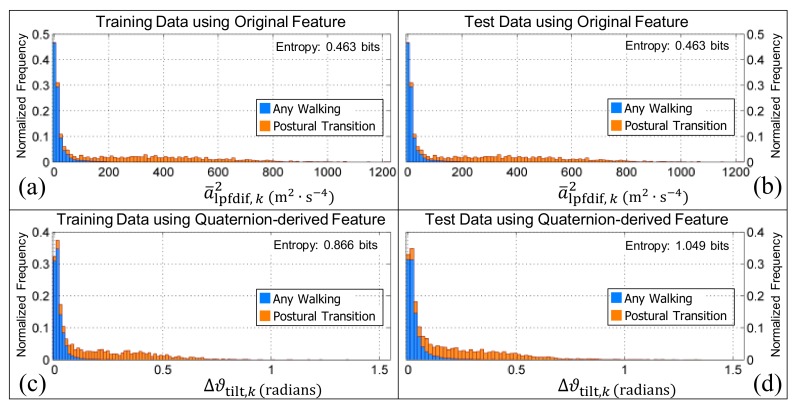
The normalized histograms of the walking (including walking up or down) and postural transition classes (illustrated in Figure 4) for the features ${\bar{a}}_{\mathrm{lpfdif},k}^{2}$ and $\Delta {\bar{\vartheta }}_{\mathrm{tilt},k}$. Panels (**a**,**c**) are the histograms obtained when the training data are used (i.e., the data from the younger and older cohorts, respectively). Panels (**b**,**d**) are the histograms obtained when the test data are used (i.e., the data from the younger and older cohort after it had been re-oriented with each of the quaternions in Figure 1b–f). The bar charts in all panels are ‘stacked’. Note that the Shannon entropy for the original feature is consistently lower than the quaternion-derived feature which suggests that it will be better at distinguishing the activity classes.

Whilst the new feature, ${\bar{\vartheta }}_{\mathrm{tilt},k}$, appears to improve the recognition rate of both standing and sedentary periods of activity, using the change in the shortest rotation between the upward and average orientations, $\Delta {\bar{\vartheta }}_{\mathrm{tilt},k}$ to distinguish between postural transitions and periods of walking (i.e., walking upstairs, walking downstairs, or walking on a level surface) does not. This is evident by the increase in Shannon entropy (when ${\bar{\vartheta }}_{\mathrm{tilt},k}$ is compared to $\Delta {\bar{\vartheta }}_{\mathrm{tilt},k}$) whether or not the feature values are generated from the training or testing data, i.e., from 0.463 bits to 0.866 bits, or from 0.463 bits to 1.049 bits, respectively (see Figure 8). Additionally, since the Shannon entropy of ${\bar{a}}_{\mathrm{lpfdif},k}^{2}$ remains constant at 0.463 bits irrespective of the dataset used, it confirms that the feature previously described [56] is orientation invariant, as expected.

**Figure 9:**
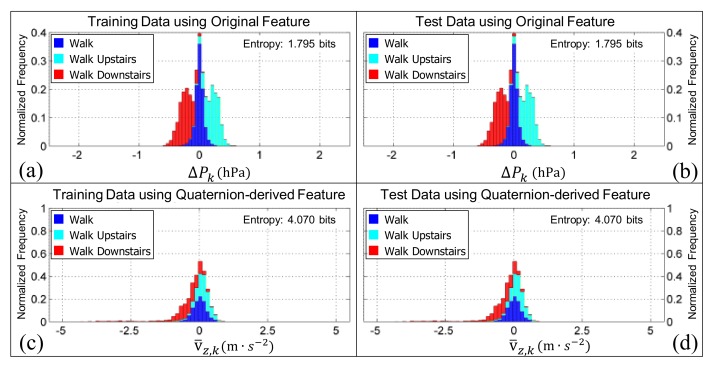
The normalized histograms of the walk, walking upstairs, and walking downstairs classes for the features $\Delta P_{k}^{}$ and ${\bar{v}}_{z,k}^{}$. Panels (**a**,**c**) are the histograms obtained when the training data are used (i.e., the data from the younger and older cohorts, respectively). Panels (**b**,**d**) are the histograms obtained when the test data are used (i.e., the data from the younger and older cohort after it had been re-oriented with each of the quaternions in Figure 1b–f). The bar charts in all panels are ‘stacked’. Note how the Shannon entropy of each feature remains constant whether it is computed from the training or test data (which, remember, is the training data re-oriented), confirming that both features are invariant to the initial orientation, as expected.

Conversely, both the average differential pressure, $\Delta P_{k}^{}$, and the velocity in the vertical direction of the estimated GFR, ${\bar{v}}_{z,k}^{}$, are orientation invariant as evident by the Shannon entropy which remains constant whether or not the training or testing data are used, for both the original feature (Figure 9a,b) and the quaternion-derived feature (Figure 9c,d). The Shannon entropy of $\Delta P_{k}^{}$ (1.795 bits) is substantially smaller than ${\bar{v}}_{z,k}^{}$ (4.070 bits) which suggests that the estimated velocity in the vertical direction (obtained by fusing vertical acceleration and barometric pressure using an extended Kalman filter) of the estimated GFR is not as useful in distinguishing between walking on flat or inclined surfaces when compared to using the rate of change of pressure measured by the barometric altimeter alone.

Although speculative, it is likely that large amplitude accelerations measured by the IMU in the pants pocket during walking is masking the subtle pattern changes in vertical acceleration associated with ascending/descending stairs. It is plausible that if the accelerometer had been placed in a chest pocket, an improved estimate of vertical acceleration may have been obtained by the Kalman filter.

### 7.2. Comparing the Overall Performance of Models for HAR

When the HMHA is trained and tested with data from the cohort of younger adults (at the original sampling rate: $f_{\mathrm{IMU}}$ = 100 Hz; $f_{\mathrm{BAR}}$ = 16 Hz), or trained with the older cohort, and tested on the data from the younger cohort after it has been re-oriented, the performance improvement of the models (i.e., the 95% confidence interval of the Cohen’s kappa, $\kappa_{{\mathrm{CI}95}^{}}$, and total class sensitivity, $\varrho_{{\mathrm{CI}95}^{}}$) are negligible. For two out of the three remaining scenarios, there are substantial improvements in the model’s performance when the quaternion-derived features developed herein (i.e., ${}^{g}{\bar{\omega }}_{xy,k}^{2}$ and ${\bar{\vartheta }}_{\mathrm{tilt},k}$) are incorporated into the process of human activity recognition. This can be observed in Table 3 when the model is trained with data from the older cohort and tested with the data from the older cohort after it has been re-oriented (i.e., $\kappa_{{\mathrm{CI}95}^{}}$ increases from $\left[0.685,0.697\right]$ to $\left[0.721,0.733\right]$; $\varrho_{{\mathrm{CI}95}^{}}$ increases from $\left[77.6\%,78.5\%\right]$ to $\left[79.9\%,80.7\%\right]$), as well as when the model is trained with data from both cohorts and tested with the data from both cohorts after it has been re-oriented (i.e., $\kappa_{{\mathrm{CI}95}^{}}$ increases from $\left[0.702,0.713\right]$ to $\left[0.732,0.742\right]$; $\varrho_{{\mathrm{CI}95}^{}}$ increases from $\left[78.4\%,79.2\%\right]$ to $\left[80.3\%,81.1\%\right]$).

It is particularly noteworthy that the performance of the model trained with the ‘Best Features’ using the data collected from the younger cohort and tested with the ‘best beatures’ using the data collected from the older adults after it has been re-oriented (i.e., $\kappa_{{\mathrm{CI}95}^{}}$ = $\left[0.782,0.793\right]$; $\varrho_{{\mathrm{CI}95}^{}}$ = $\left[84.9\%,85.6\%\right]$) is comparable to the performance of the model trained with the data collected from the older cohort and tested with the data from the younger cohort (i.e., $\kappa_{{\mathrm{CI}95}^{}}$ = $\left[0.765,0.784\right]$; $\varrho_{{\mathrm{CI}95}^{}}$ = $\left[82.3\%,83.7\%\right]$). This contradicts the finding of our previous work [56] in which the performance of a model for HAR trained on younger cohorts degraded substantially when tested on older cohorts (due to the use of the tilt angle feature, ${\bar{\Theta }}_{\mathrm{tilt},k}^{}$, which was not orientation invariant (see Table 3, the column labeled ‘Original Features’)), compared to the opposite scenario in which the model is trained with the older cohort’s data and tested with the data from the younger cohort, which gives the better performance.

**Figure 10:**
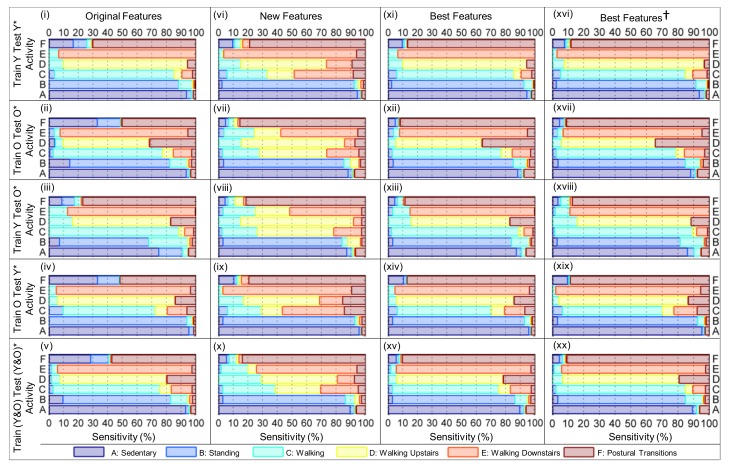
The column titled: ‘Original Features’ (i.e., panels (**i**–**v**)) correspond to hierarchical models of human activity that were trained and tested with the features developed in our previous work [56] using the hierarchical model of human activity (HMHA) illustrated in Figure 4b; ‘New Features’ (i.e., panels (**vi**–**x**)) correspond to hierarchical models of human activity that were trained and tested with the features developed herein using the HMHA illustrated in Figure 4c; ‘Best Features’ (i.e., panels (**xi**–**xv**)) correspond to hierarchical models of human activity that were trained and tested with a combination of the original and New Features using the HMHA illustrated in Figure 4d; ‘Best Features${}^{†}$’ (i.e., panels (**xvi**–**xx**)) equivalent to ‘Best Features’ with the IMU data re-sampled to 40 Hz, the barometer data to 20 Hz.

The improvements in total classification sensitivity and Cohen’s kappa gained by incorporating the quaternion-derived features (see Table 3 column labeled ‘Best Features’) persist when the data from the IMU are re-sampled at a reduced rate (see the column labeled ‘Best Features ${}^{†}$’ in Table 3). This demonstrates the robustness of both the features and the HMHA to a decrease in the sample rate, which is an important design consideration given the limited battery life of wearable sensors (i.e., smartphones, smartwatches, etc.). Interestingly, there are marginal improvements in Cohen’s kappa (i.e., from κ = $\left[0.732,0.742\right]$ when $f_{\mathrm{IMU}}$ = 100 Hz to κ = $\left[0.778,0.787\right]$ when $f_{\mathrm{IMU}}$ = 40 Hz) and total class sensitivity (i.e., from $\varrho_{\mathrm{CI}95}$ = $\left[80.3\%,81.1\%\right]$ when $f_{\mathrm{IMU}}$ = 100 Hz to $\varrho_{\mathrm{CI}95}$ = $\left[84.1\%,84.8\%\right]$ when $f_{\mathrm{IMU}}$ = 40 Hz) when the model is trained with the data from the younger and older cohorts and tested with the data from the younger and older cohorts after it has been re-oriented. Upon analyzing the class sensitivity of these two hierarchical models of human activity, it is evident that this is primarily due to an increase in the sensitivity of detecting the walking class, from ~72% to ~82% (see Figure 10xv,xx). This improvement can be attributed to the use of the quaternion derived feature, ${}^{g}{\bar{\omega }}_{xy,k}^{2}$ which measures the amount of pitch/roll rotation in the estimated GFR, a more consistent frame of reference than the local sensor frame.

### 7.3. Identifying Which Features Drive Model Performance

When Figure 10i,xi are compared (i.e., a HMHA trained with the Original Features extracted from the younger cohort and a HMHA trained with the Best Features extracted from the younger cohort), the differences in the model’s performance become apparent. Most notably, the sensitivity for the postural transition class increased from 70.05% to 86.64%. This improved performance is a by-product of modest increases in the model’s ability to identify standing (i.e., the standing class sensitivity increased from 88.29% to 89.98%) and sedentary periods of activity (i.e., the class sensitivity increased from 93.67% to 94.42%). These pieces of evidence support the argument that the quaternion-derived feature, ${}^{g}{\bar{\omega }}_{xy,k}^{2}$, is better at distinguishing periods of activity (i.e., walking, or postural transitions) from periods of inactivity (i.e., standing or sedentary), a trend which is consistent across each of the five training and testing scenarios proposed in Section 6. However, it is likely that ${}^{g}{\bar{\omega }}_{xy,k}^{2}$ would not be as effective in this task if the smartphone is placed in the user’s chest pocket, which pitches and rolls less when compared to the thigh (i.e., it rotates less about the *x* and *y* axis of the estimated GFR) during walking.

The underlying causes for the improvement in the HMHA become clear after analyzing the sensitivity of the activity classes listed in Figure 10. When the columns corresponding to the HMHAs built using the Original Features and New Features (e.g., when Figure 10i is compared to Figure 10vi, Figure 10ii to Figure 10vii, and so on) were compared, it is evident that the model’s sensitivity to periods of walking upstairs decreased dramatically (e.g., in the case of (i) and (v), from 84.21% to 58.16%) when the differential pressure, $\Delta P_{k}$, was replaced with the moving average velocity in the vertical direction of the estimated GFR, ${\bar{v}}_{z,k}$. This persisted whether the data from the younger or older cohort was used (i.e., when the columns entitled ‘Original Features’ and ‘New Features’ of Figure 10 are compared, the model’s sensitivity to periods of walking either upstairs or downstairs is reduced). $\Delta P_{k}$ is superior to ${\bar{v}}_{z,k}$ in estimating vertical velocity and hence walking on stairs.

On the other hand, when ${\bar{\Theta }}_{\mathrm{tilt},k}$ was substituted with ${\bar{\vartheta }}_{\mathrm{tilt},k}$, the sensitivity of the model to standing classes increases (from 60–70% to >80%) when the HMHA was trained with: (a) the older cohort’s data and tested with the older cohort’s data after it had been re-oriented (the second row in Figure 10); (b) the younger cohort’s data and tested with the older cohort’s data after it had been re-oriented (the fourth row in Figure 10); (c) the data from both cohorts and tested with the data from both cohorts after it had been re-oriented. This improvement underscores the utility of learning the orientation of the device when the body is definitely upright (i.e., when walking), demonstrating how this method can intuitively account for the variability in sensor measurements which may arise due to inconsistent device orientation when the IMU is placed on the body.

In addition, the rate of misclassification of sedentary and stationary periods of activity as postural transitions decreases markedly. This phenomena is consistent across the five scenarios evaluated (recall Section 6). When the columns labeled ‘Original Features’ and ‘Best Features’ are compared row by row, periods of standing that were originally classified as postural transitions are all but eliminated (e.g., compare Figure 10i and Figure 10xi), whilst the misclassification rate of sedentary activity as postural transitions decreased from ~16% to ~9% (compare Figure 10i and Figure 10xi); ~32% to ~5% (compare Figure 10ii and Figure 10xii); ~9% to ~5% (compare Figure 10iii and Figure 10xiii); ~33% to ~10% (compare Figure 10iv and Figure 10xiv); ~29% to ~7% (compare Figure 10v and Figure 10xv).

### 7.4. Comparing Model Performance at Different Sampling Rates

Due to the limited battery life of smartphones, it is becoming increasingly important that algorithms for human activity recognition are able to operate at a reduced sampling rate without suffering a degradation in classification accuracy. Consequently, the robustness of the model’s developed with the ‘Best Features’ were evaluated by training the model with the data collected from the younger and/or older cohort at 100 Hz, and testing the model’s performance with data from the younger and/or older cohort at 40 Hz after it had been virtually re-oriented (and vice versa). From Table 4 it is evident that both the Cohen’s kappa, and total class sensitivity of the HMHA proposed in Figure 4d remain consistent (i.e., the 95% confidence intervals overlap for almost all of the training and testing combinations evaluated) whether or not the HMHA is trained with the data at 100 Hz (i.e., the higher sampling rate) and tested with the re-oriented data at 40 Hz (i.e., the reduced sampling rate), or the reciprocal scenario in which the HMHA is trained with the data at 40 Hz and tested with the re-oriented data at 100 Hz.

The sole exception to this trend is the scenario in which the data re-sampled at 40 Hz from both the younger and older cohorts are used to train the HMHA, whilst the data sampled at 100 Hz from the younger and older cohorts after it has been re-oriented are used to test the HMHA. In this particular scenario, the ninety-five percent confidence interval of the Cohen’s kappa, κ, increased by ~0.04 from $\kappa_{{\mathrm{CI}95}^{}}$ = $\left[0.732,0.742\right]$ to $\kappa_{{\mathrm{CI}95}^{}}$ = $\left[0.776,0.786\right]$. Similarly, the ninety-five percent confidence interval of the total class sensitivity increased by ~3% from $\varrho_{{\mathrm{CI}95}^{}}$ = $\left[80.3\%,81.1\%\right]$ to $\varrho_{{\mathrm{CI}95}^{}}$ = $\left[84.0\%,84.7\%\right]$ (see the bottom row of Table 4).

**Figure 11:**
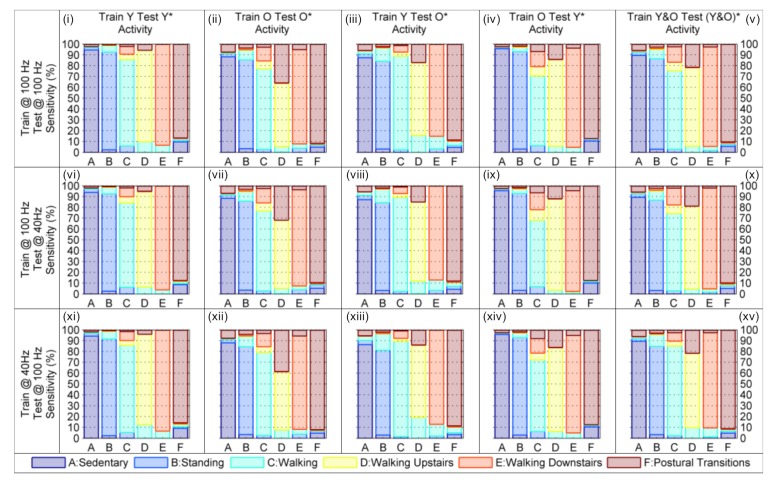
Class Sensitivity for a Hierarchical Model of Human Activity Recognition using the Best Features: (**i**–**v**) trained with the IMU data at 100 Hz and tested with the IMU data at 100 Hz after it has been re-oriented; (**vi**–**x**) trained with the IMU data at 100 Hz and tested with the IMU data at 40 Hz after it has been re-oriented; (**xi**–**xv**) trained with the IMU data at 40 Hz and tested with the IMU data at 100 Hz after it has been re-oriented.

After analyzing Figure 11 it is evident that the model’s sensitivity to each activity remains relatively consistent as long as only one of the cohort’s data is used to train the model, and the other cohort’s data is used to test the model, irrespective of the sampling rate (i.e., when Figure 11iii,viii,xiii are compared; Figure 11iv,ix,xiv are compared, and so on). When both cohort’s data are used (i.e., when Figure 11v,x,xv are compared), the sensitivity of the model to the sedentary, standing, and postural transition classes is remarkably consistent whilst the sensitivity of the model to the three different walking, classes varies (whether or not the HMHA is trained with the data re-sampled at 40 Hz or the data sampled at 100 Hz). From Table 5 it is easy to see that this robustness in performance can be attributed to the relatively constant threshold values of ${}^{g}{\bar{\omega }}_{xy,k}^{2}$, and ${\bar{\vartheta }}_{\mathrm{tilt},k}$ which change by <0.1 (rad${}^{2}$·s${}^{-2}$ and radians, respectively), suggesting that these features are robust to both the variation in sampling rate and the cohort from which the threshold is extracted (i.e., the threshold changes little whether trained on the younger and/or older cohort’s data).

Interestingly, the recognition rate of the postural transition class also remains fairly consistent (i.e., between ~89–91%), irrespective of the data which are used to train and test the HMHA. This suggests that ${\bar{a}}_{\mathrm{lpfdif},k}^{2}$ is also robust to variations in the sampling rate of the IMU and the cohort from which the threshold are determined (see Table 5).

In the case of the walking, walking upstairs, and walking downstairs classes, the differences were negligible when the HMHA was trained with the data sampled at 100 Hz and tested with the same data after it had been re-oriented; or trained with the data at 100 Hz and tested with the data re-sampled at 40 Hz after it had been re-oriented. However, when the model was trained with the data re-sampled at 40 Hz and tested with the data at 100 Hz there were slight changes in the class sensitivity when compared to either of the two previously mentioned scenarios.

In particular, the model’s sensitivity to the walking class increased from ~71–72% to ~82% (trained with data at 40 Hz, tested with data at 100 Hz) due to marked reductions in periods of walking upstairs and walking downstairs being incorrectly identified as walking on a level surface (i.e., from 7.95% to 4.5% and 15.32% to 7.82%, respectively). Similarly, the sensitivity of the walking upstairs class decreased from ~76% to ~68% (see Appendix C, the row labeled ‘Train Y&O Test (Y&O)${}^{*}$’ in Table A2) due to the increased misclassification of periods of walking upstairs as periods of walking on a level surface (i.e., from 4–5% to ~10%; see Figure 11 and compare panels (x) and (xv)). This suggests that the smaller threshold of $\Delta P_{k}$ = 0.092 hPa·s${}^{-1}$ (see Table 5) is better (when compared to $\Delta P_{k}$ = 0.119 hPa·s${}^{-1}$) at distinguishing between periods of walking on a level surface versus walking upstairs.

This trend was mirrored in the reduction of the hierarchical model’s sensitivity to the walking downstairs class; i.e., decreasing from ~91–92% to ~87% (see Appendix C, the row labeled ‘Train Y&O Test (Y&O)${}^{*}$’ in Table A2) due to the increased misclassification of periods of walking downstairs as periods of walking on a level surface (i.e., from 3–4% to ~8%; see Figure 11 and compare panels (x) and (xv)). Again, this suggests that the threshold of $\Delta P_{k}$ = −0.062 hPa·s${}^{-1}$ (see Table 5) is better than the threshold of $\Delta P_{k}$ = −0.094 hPa·s${}^{-1}$ (a change of ~51% in the threshold value) at distinguishing between periods of walking on a level surface versus walking downstairs.

### 7.5. Comparison to the State-of-the-Art

In order to draw a fair comparison with other published work that is representative of state-of-the-art methods, the scope of these comparisons is limited to reports which only utilized the smartphone’s internal sensing components to classify human activity. With this in mind, the state-of-the-art deep learning methods (recall Section 1.3.3) proposed by Ordoñez et al. [45] and Li et al. [78] are excluded because they utilize measurements from multiple IMUs that are placed at different anatomical locations on the body, whilst the works of Ravi et al. [43] and Ronao and Cho et al. [42] are included. Similarly, the ‘feature engineering and classification’-based approaches (recall Section 1.3.2) developed by Bao and Intille et al. [79] are omitted, whilst the works of Anguita et al. [80] and Shoaib et al. [29] are included.

Anguita et al. developed a hardware-friendly multi-class support vector machine which processed the accelerometer and gyroscope data (at 50 Hz) from a waist-worn smartphone (i.e., attached to a belt worn about their waist) to identify activities of daily living in a cohort of thirty participants aged between nineteen and 48 years. From these six channels, they extracted 561 spatial or spectral features (every 1.25 seconds using 50% overlapping windows) to identify six activities with a sensitivity between 72% and 96%: walking (95.6%), walking upstairs (72.1%), walking downstairs (79.7%), standing (92.2%), sitting (96.4%), and lying (100%) [80].

Shoaib et al. evaluated the utility of a smartphone’s internal sensors for the purposes of human activity recognition. They studied ten male participants, aged between 25 and 30 years, whilst a smartphone was firmly fixed to their body with a strap at one of five positions on their body (right and left front jeans pocket, on a belt near the right hip, right wrist, right upper arm). A smartphone application recorded the accelerometer, gyroscope, and magnetometer data at 50 Hz whilst each participant performed six activities of daily living (walking, jogging, sitting, standing, biking, walking upstairs, and walking downstairs) [29]. When features were extracted every two seconds (with 50% overlapping windows), the gyroscope-based features proved most effective in identifying periods of walking upstairs and walking downstairs (particularly when the sensor was placed in the jeans pocket or on the belt), whilst features from the magnetometer should only be used if they are independent of heading. Moreover, they advocate against ‘blindly combining different sensors’, suggesting a more manual approach to system and feature design.

Deep learning approaches attempt to tease out more subtle differences, imperceptible by human observation, in wearable sensor data which can be used for the purposes of HAR. Ronao and Cho, recruited 30 participants (age range not disclosed) to evaluate the performance of a model for HAR, based on deep convolutional neural networks (convnet). The smartphone was placed in a pocket of the participants’ clothing (location on body not disclosed), whilst data from the accelerometer and gyroscope were recorded at 50 Hz [42]. When the data were segmented in 2.5 second intervals with 50% overlap, the convnet could identify six activities: walking (98.99%), walking upstairs (100.00%), walking downstairs (100.00%), standing (93.23%), sitting (88.88%), lying (87.71%); with an overall sensitivity of 94.79%. Before the accelerometer and gyroscope data could be processed, each channel (of six) needed to be normalized by subtracting the mean of each signal, and dividing each channel by the channel’s standard deviation. At this point, 2.5 second data segments are input to a five-layer convnet comprised of three convolutional/pooling layers (with 96, 192, 192 neurons in each layer, respectively), a fully connected layer comprised of 1000 neurons, and a softmax classification layer with six neurons.

Ravi et al. combined features extracted from the spectrogram (i.e., the short-time Fourier transform coefficients) of accelerometer and gyroscope signals (both of which sampled at either 50 Hz or 200 Hz, respectively) with a three layered network comprised of a temporal convolution layer (15 filters, 80 nodes), fully-connected layer, and soft-max classification layer for the purposes of HAR. Data was obtained from ten subjects (using five different smartphones) who were allowed to place the phone anywhere on their body (or in their hand/bag) whilst they performed six activities of daily living. The total class sensitivity of their model for HAR was 95.7% with class sensitivities of ~95% (running), ~95% (walking), ~96% (cycling), ~96% (casual movement), ~96% (public transport), ~98% (idle), and ~74% (standing). Whilst the features derived from the six channel spectrogram enabled highly-variable activities to be distinguished from repetitive activities, the absence of time-domain-based features limited the model’s ability to infer the user’s postural orientation, which was further limited by the fact that the phone could be placed at various parts of the body, in the hand, or in a bag [43].

The model for HAR constructed by Gu et al. [81] implemented denoising autoencoders (two layers, 1000 neurons per layer) combined with a softmax classification layer to automate the HAR process. Features were extracted from two-second intervals of data from the smartphone’s accelerometer, gyroscope, magnetometer, and barometer (all of which were sampled at 64 Hz, except for the barometer which was sampled at 32 Hz). Twelve participants (six male, six female) aged between twenty-five and thirty-five years were recruited to train the model to recognize eight activities of daily living. When the data from all four sensors were used by the denoising autoencoders (corrupting noise level = 0.5, learning rate = 1 × 10${}^{-3}$, weight of sparsity penalty term = 1), the F-measure is 94.04% and the class sensitivity for the eight activities are: stationary (~98%), walking (~92%), stationary but using the phone (~96%), running (~97%), walking upstairs (~94%), walking downstairs (~93%), elevator up (~84%), elevator down (~87%).

A general pitfall of all of the above deep learning approaches is that this approach does not inherently allow the training of the neural network to be constrained by the domain knowledge that the smartphone could be placed anywhere on the body and with any orientation. For deep learning approaches, some safeguards against obtaining a classifier model which is not robust to such variability in smartphone placement and orientation involves collecting large datasets which capture this variability, or to perform preprocessing of the smartphone signals to generate features which are tolerant to such variability; the latter somewhat goes against the spirit of the deep learning approach.

## 8. Limitations

There are limitations with the study presented herein which need to be acknowledged. The model for HAR developed is dependent on the wearable sensor (i.e., device containing an IMU and barometer, such as a smartphone) remaining in the pants pocket throughout the day, which is not a realistic expectation since the individual’s lower body garments may not always have a suitable pocket, or a pocket large enough to place the wearable sensor. If the wearable sensor is strapped to the thigh, the quaternion-derived feature, ${\bar{\vartheta }}_{\mathrm{tilt},k}$, should always be able to separate standing and sedentary periods. If the device is sporadically removed from the pants pocket whilst the person is moving, it is conceivable that the walking detector (Equation (13)) could ‘learn’ an incorrect upright orientation, $q_{\mathrm{upright},k}^{}$, thereby reducing the accuracy of the model for HAR until it relearns the correct upright orientation from the next 2.5 s of true walking data; robustness to this scenario will be evaluated in future work.

## 9. Conclusions and Future Work

This paper developed a model for HAR capable of recognizing six human activities (standing, sedentary, walk, walk upstairs, walk downstairs, as well as postural transitions between the standing and sedentary classes), regardless of the smartphone’s orientation in the pants pocket by using a quaternion-based complementary filter [63] to estimate the device’s orientation, thereby enabling sensor measurements to be expressed in a consistent frame of reference (the world/global frame). Four New Features were developed, and two were shown to be useful in the classification of human activities, namely ${}^{g}{\bar{\omega }}_{xy,k}^{2}$, which utilized an estimate of the IMU’s orientation to determine the magnitude of the pitch/roll angular velocity, and ${\bar{\vartheta }}_{\mathrm{tilt},k}^{}$, which measured the angle between the recent average orientation and the estimated upright orientation; upright orientation was estimated as the average orientation of the IMU when walking was detected. The success of these quaternion-derived features suggest that existing methods for recognizing human activities would benefit from converting all measurements to the global frame of reference where the feature values would be more consistent, especially if the orientation of the IMU with respect to the body is not fixed.

## Figures and Tables

**Table 1:** Tuning parameters of the computationally-efficient adaptive error-state kalman filter.

Sampling Rate	${}^{†}\;\sigma_{G}^{}$	$c_{a}^{}$	$c_{m}^{}$	${}^{‡}$ $N_{\mathrm{short}}^{}$	${}^{‡}$ $N_{\mathrm{long}}^{}$	${}^{⋆}$ $\xi_{a}^{}$	${}^{‡}$ $N_{m}^{}$	${}^{⋆⋆}$ $\xi_{\mathrm{xy}}^{}$
$f_{s}^{}$ = 100 Hz	0.01	0.1	0.99	7	49	1	7	5
$f_{s}^{}$ = 40 Hz	0.01	0.1	0.99	3	19	1	3	5

${}^{†}$ rad/s; ${}^{‡}$ samples; ${}^{⋆}$ m s ^−2^; ⋆⋆ normalized units/s.

**Table 2:** Features extracted from the accelerometer, gyroscope, and barometric altimeter.

No.	Feature	Description
(1)	${\bar{\omega }}_{\mathrm{bpf},k}^{2}$ $=\frac{1}{N}\sum_{j=i}^{k}{\left(\omega_{\mathrm{bpf},x}^{2}+\omega_{\mathrm{bpf},y}^{2}+\omega_{\mathrm{bpf},z}^{2}\right)}_{j}$	average squared band-pass-
		filtered angular velocity
(2)	${\bar{\Theta }}_{\mathrm{tilt},k}^{}$ $=\frac{1}{N}\sum_{j=i}^{k}\cos^{-1}{\left(a_{\mathrm{lpf},y}^{}\;/\;\sqrt{\left(a_{\mathrm{lpf},x}^{2}+a_{\mathrm{lpf},y}^{2}+a_{\mathrm{lpf},z}^{2}\right)}\right)}_{j}^{†}$	average inclination angle
(3)	${\bar{a}}_{\mathrm{lpfdif},k}^{2}$ $=\frac{1}{N}\sum_{j=i}^{k}{\left(a_{\mathrm{lpfdif},x}^{2}+a_{\mathrm{lpfdif},y}^{2}+a_{\mathrm{lpfdif},z}^{2}\right)}_{j}$	average squared band-
		pass-filtered acceleration
(4)	$\Delta P_{k}^{}$ $=\frac{1}{N}\sum_{j=i}^{k}\partial p_{j}^{}$	average differential pressure
(5)	${}^{g}{\bar{\omega }}_{xy,k}^{2}$ $=\frac{1}{N}\sum_{j=i}^{k}{{(}^{g}\omega_{x}^{2}{+}^{g}\omega_{y}^{2})}_{j}$	average squared pitch/roll
		angular velocity
(6)	${\bar{\vartheta }}_{\mathrm{tilt},k}$ $=\frac{1}{N}\sum_{j=i}^{k}\vartheta_{\mathrm{tilt},j}^{}$	average of the shortest rotation between
		the upright and average orientations
(7)	$\Delta {\bar{\vartheta }}_{\mathrm{tilt},k}^{}$ $=|{\bar{\vartheta }}_{\mathrm{tilt},k}^{}-{\bar{\vartheta }}_{\mathrm{tilt},k-N}^{}|$	change in the shortest rotation between
		the upward and average orientations
(8)	${\bar{v}}_{z,k}^{}$ $=\frac{1}{N}\sum_{j=i}^{k}{\dot{z}}_{j}^{}$	average velocity in the vertical
		direction of the estimated GFR

Note: *i* = *k*−*N* +1; *N*–the number of samples in a 2.5 s analysis window (⌊2.5×$f_{\mathrm{IMU}}^{}\rfloor$); The variables: $\omega_{\mathrm{bpf}}^{}$, $a_{\mathrm{lpfdif}}^{}$, $a_{\mathrm{lpf}}^{}$, and ∂p correspond to the filtered signals described in [56]; ${}^{†}$ The tilt angle [48].

**Table 3:** Ninety-five percent confidence intervals for the Cohen’s Kappa and total class sensitivity when hierarchical models for human activity recognition were developed with different features.

Cohen’s Kappa ($\kappa_{{\mathrm{CI}95}^{}}$)					
Train	Test	Original Features	New Features	Best Features	Best Features ${}^{†}$
Y	Y ${}^{⋆}$	$\left[0.820,0.837\right]$	$\left[0.606,0.629\right]$	$\left[0.827,0.844\right]$	$\left[0.824,0.841\right]$
O	O ${}^{⋆}$	$\left[0.685,0.697\right]$	$\left[0.478,0.491\right]$	$\left[0.721,0.733\right]$	$\left[0.730,0.741\right]$
Y	O ${}^{⋆}$	$\left[0.675,0.688\right]$	$\left[0.468,0.481\right]$	$\left[0.782,0.793\right]$	$\left[0.779,0.790\right]$
O	Y ${}^{⋆}$	$\left[0.760,0.780\right]$	$\left[0.590,0.613\right]$	$\left[0.765,0.784\right]$	$\left[0.761,0.781\right]$
Y&O	(Y&O)${}^{⋆}$	$\left[0.702,0.713\right]$	$\left[0.544,0.556\right]$	$\left[0.732,0.742\right]$	$\left[0.778,0.787\right]$
**Total Class Sensitivity ($\varrho_{{\mathrm{CI}95}^{}}$%)**					
**Train**	**Test**	**Original Features**	**New Features**	**Best Features**	**Best Features ${}^{†}$**
Y	Y ${}^{⋆}$	$\left[86.7,88.0\right]$	$\left[69.0,70.8\right]$	$\left[87.2,88.5\right]$	$\left[87.0,88.3\right]$
O	O ${}^{⋆}$	$\left[77.6,78.5\right]$	$\left[57.3,58.4\right]$	$\left[79.9,80.7\right]$	$\left[80.6,81.5\right]$
Y	O ${}^{⋆}$	$\left[77.7,78.6\right]$	$\left[56.3,57.3\right]$	$\left[84.9,85.6\right]$	$\left[84.8,85.5\right]$
O	Y ${}^{⋆}$	$\left[82.0,83.5\right]$	$\left[67.5,69.3\right]$	$\left[82.3,83.7\right]$	$\left[82.0,83.5\right]$
Y&O	(Y&O)${}^{⋆}$	$\left[78.4,79.2\right]$	$\left[63.9,64.8\right]$	$\left[80.3,81.1\right]$	$\left[84.1,84.8\right]$

${}^{⋆}$ Test data were obtained by re-orienting the data from the younger (Y) and/or older (O) cohort (see Section 3.1); ${}^{†}$ IMU data were re-sampled at 40 Hz, barometer data were re-sampled at 20 Hz.

**Table 4:** Ninety-five percent confidence intervals for the Cohen’s Kappa and total class sensitivity (%) when a hierarchical model of human activity (HMHA) was developed with the Best Features at different sampling rates.

		Cohen’s Kappa ($\kappa_{{\mathrm{CI}95}^{}}$)			Total Class Sensitivity ($\varrho_{{\mathrm{CI}95}^{}}$%)		
Train		100 Hz	100 Hz	40 Hz ${}^{†}$	100 Hz	100 Hz	40 Hz ${}^{†}$
	Test ${}^{⋆}$	100 Hz	40 Hz ${}^{†}$	100 Hz	100 Hz	40 Hz ${}^{†}$	100 Hz
Y	Y ${}^{⋆}$	$\left[0.827,0.844\right]$	$\left[0.819,0.837\right]$	$\left[0.823,0.841\right]$	$\left[87.2,88.5\right]$	$\left[86.6,87.9\right]$	$\left[87.0,88.3\right]$
O	O ${}^{⋆}$	$\left[0.721,0.733\right]$	$\left[0.720,0.732\right]$	$\left[0.728,0.740\right]$	$\left[79.9,80.7\right]$	$\left[79.8,80.6\right]$	$\left[80.5,81.3\right]$
Y	O ${}^{⋆}$	$\left[0.782,0.793\right]$	$\left[0.786,0.796\right]$	$\left[0.777,0.788\right]$	$\left[84.9,85.6\right]$	$\left[85.2,85.9\right]$	$\left[84.6,85.4\right]$
O	Y ${}^{⋆}$	$\left[0.765,0.784\right]$	$\left[0.752,0.772\right]$	$\left[0.769,0.789\right]$	$\left[82.3,83.7\right]$	$\left[81.3,82.8\right]$	$\left[82.6,84.1\right]$
Y&O	(Y&O) ${}^{⋆}$	$\left[0.732,0.742\right]$	$\left[0.726,0.736\right]$	$\left[0.776,0.786\right]$	$\left[80.3,81.1\right]$	$\left[79.9,80.6\right]$	$\left[84.0,84.7\right]$

${}^{⋆}$ Test data were obtained by re-orienting the data from the younger (Y) and/or older (O) cohort (see Section 3.1); ${}^{†}$ IMU data were re-sampled at 40 Hz, barometer data were re-sampled at 20 Hz.

**Table 5:** Comparison of thresholds when HMHA recognition were developed with the Best Features at different sampling rates.

Training Data	Feature	Threshold		Rule *
		100 Hz	40 Hz	
Y	${}^{g}{\bar{\omega }}_{xy,k}^{2}$	0.232	0.195	Inactive${}^{†}$ if ${}^{g}{\bar{\omega }}_{xy,k}^{2}$ ≤ threshold, else Active${}^{‡}$
O		0.249	0.237	
Y& O	(rad${}^{2}$·s${}^{-2}$)	0.230	0.202	
Y	${\bar{\vartheta }}_{\mathrm{tilt},k}$	0.668	0.689	Standing if ${\bar{\vartheta }}_{\mathrm{tilt},k}$ ≤ threshold, else Sedentary (i.e., sitting or lying)
O		0.616	0.617	
Y& O	(radians)	0.640	0.617	
Y	${\bar{a}}_{\mathrm{lpfdif},k}^{2}$	75.6	93.4	Any Walking${}^{§}$ if ${\bar{a}}_{\mathrm{lpfdif},k}^{2}$ ≤ threshold, else Postural Transition
O		36.4	32.8	
Y& O	(m${}^{2}$·s${}^{-4}$)	46.7	46.4	
Y	$\Delta P_{k}^{}$	−0.107	−0.101	Walking Downstairs if $\Delta P_{k}^{}$ ≤ threshold, else Walking
O		−0.067	−0.068	
Y& O	(hPa·s${}^{-1}$)	−0.062	−0.094	
Y	$\Delta P_{k}^{}$	0.128	0.142	Walking if $\Delta P_{k}^{}$ ≤ threshold, else Walking Upstairs
O		0.092	0.105	
Y& O	(hPa·s${}^{-1}$)	0.092	0.119	

Inactive${}^{†}$ — any of the standing or sedentary (sitting/lying) classes; active${}^{‡}$ — any of the walking, walking upstairs, walking downstairs, or postural transition classes; any walking${}^{§}$ — either of the walking, walking upstairs, or walking downstairs classes. * Each rule corresponds to a node of the HMHA illustrated in Figure 4d.

## Appendix A. Average of Multiple Quaternions

Gramkow’s method was used to calculate the average of *N* quaternions [82]. Note: the quaternion is normalized after each component of $\bar{q}$ has been calculated (see Equation (A1)). If the scalar component of a quaternion, $q=\left[\begin{array}{cccc} q_{0}^{} & q_{1}^{} & q_{2}^{} & q_{3}^{} \end{array}\right]$, in the window was negative (i.e., if $q_{0}^{}$< 0) each component of the quaternion was negated (thereby preserving the rotational information since q and −q represent the same rotation [83]) so that each quaternion lies in the same half plane.

(A1) $$\begin{array}{c} \bar{q}=f_{q,\mathrm{avg}}^{}\left(q_{1}^{},\cdots ,q_{N}^{}\right)=\left[\begin{array}{cccc} \frac{{\bar{q}}_{0}^{}}{\left|\left|q\right|\right|} & \frac{{\bar{q}}_{1}^{}}{\left|\left|q\right|\right|} & \frac{{\bar{q}}_{2}^{}}{\left|\left|q\right|\right|} & \frac{{\bar{q}}_{3}^{}}{\left|\left|q\right|\right|} \end{array}\right]\\ {\bar{q}}_{j}^{}=\frac{1}{N}\sum_{k=1}^{N}q_{j,k}^{}\;(\mathrm{where}\;j\;\in \;\left\{0,\;1,\;2,\;3\}\right);\;\left|\left|q\right|\right|=\sqrt{{\bar{q}}_{0}^{2}+{\bar{q}}_{1}^{2}+{\bar{q}}_{2}^{2}+{\bar{q}}_{3}^{2}} \end{array}$$

## Appendix B. Shortest Rotation Between Two Quaternions

The rotation that brings two quaternions, $q_{A}^{}$ and $q_{B}^{}$, into coincidence is $q_{\mathrm{AB}}^{}$ = $q_{A}^{}⊗{\left(q_{B}^{}\right)}^{*}$, i.e., $q_{B}^{}⊗q_{\mathrm{AB}}^{}$ = $q_{A}^{}$. The shortest angle between these quaternions, 0 ≤ϑ≤π is given by $q_{\mathrm{AB},0}^{}$, the scalar component of $q_{\mathrm{AB}}^{}$, Equation (A2).

(A2) $$\begin{array}{c} \vartheta =f_{\mathrm{angle}}^{}\left(q_{A}^{},q_{B}^{}\right)=\left\{\begin{array}{c} 2\cos^{-1}(\;q_{\mathrm{AB},0}^{}),\;q_{\mathrm{AB},0}^{}\geq 0\\ 2\cos^{-1}\left(-q_{\mathrm{AB},0}^{}\right),\;q_{\mathrm{AB},0}^{}<0 \end{array}\right. \end{array}$$

## Appendix C. Ninety-five Percent Confidence Intervals for the Class Sensitivity and Class Specificity of the Hierarchical Models of Human Activity

**Table A1:** Ninety-five percent confidence intervals for the sensitivity and specificity of each activity class when HMHA were developed with different feature subsets ($f_{{\mathrm{IMU}}^{}}$ = 100 Hz; $f_{{\mathrm{bar}}^{}}$ = 16 Hz).

	Activity	Sensitivity (%)			Specificity (%)		
		Original	New	Best	Original	New	Best
		Features	Features	Features	Features	Features	Features
	Sedentary	$\left[92.8,94.5\right]$	$\left[93.6,95.2\right]$	$\left[93.6,95.2\right]$	$\left[99.3,99.6\right]$	$\left[98.4,98.9\right]$	$\left[98.4,98.9\right]$
	Standing	$\left[87.1,89.5\right]$	$\left[88.8,91.1\right]$	$\left[88.8,91.1\right]$	$\left[96.4,97.2\right]$	$\left[96.1,96.9\right]$	$\left[96.1,96.9\right]$
Train Y	Walking	$\left[79.9,82.7\right]$	$\left[25.8,28.9\right]$	$\left[78.2,81.0\right]$	$\left[94.5,95.5\right]$	$\left[97.4,98.1\right]$	$\left[96.0,96.9\right]$
Test Y *	Walking Upstairs	$\left[80.5,87.9\right]$	$\left[53.2,63.1\right]$	$\left[80.5,87.9\right]$	$\left[97.7,98.3\right]$	$\left[93.2,94.2\right]$	$\left[98.0,98.5\right]$
	Walking Downstairs	$\left[90.7,95.9\right]$	$\left[87.2,93.3\right]$	$\left[90.7,95.9\right]$	$\left[97.1,97.7\right]$	$\left[86.6,87.9\right]$	$\left[97.2,97.9\right]$
	Postural Transitions	$\left[64.0,76.1\right]$	$\left[73.4,84.2\right]$	$\left[82.1,91.2\right]$	$\left[98.0,98.6\right]$	$\left[95.3,96.1\right]$	$\left[97.8,98.3\right]$

	Sedentary	$\left[92.9,93.9\right]$	$\left[87.5,88.8\right]$	$\left[87.5,88.8\right]$	$\left[94.2,94.8\right]$	$\left[98.6,98.9\right]$	$\left[98.6,98.9\right]$
	Standing	$\left[66.9,69.2\right]$	$\left[80.9,82.7\right]$	$\left[80.9,82.7\right]$	$\left[97.2,97.5\right]$	$\left[97.2,97.5\right]$	$\left[97.2,97.5\right]$
Train O	Walking	$\left[73.6,75.0\right]$	$\left[24.5,25.8\right]$	$\left[73.4,74.8\right]$	$\left[95.1,95.7\right]$	$\left[96.6,97.1\right]$	$\left[95.9,96.5\right]$
Test O *	Walking Upstairs	$\left[54.4,62.7\right]$	$\left[66.0,73.8\right]$	$\left[54.2,62.5\right]$	$\left[96.4,96.7\right]$	$\left[81.4,82.2\right]$	$\left[96.4,96.8\right]$
	Walking Downstairs	$\left[83.9,89.8\right]$	$\left[47.9,56.7\right]$	$\left[83.9,89.8\right]$	$\left[94.0,94.5\right]$	$\left[90.3,90.9\right]$	$\left[94.0,94.5\right]$
	Postural Transitions	$\left[47.2,53.2\right]$	$\left[83.3,87.5\right]$	$\left[89.9,93.3\right]$	$\left[96.3,96.7\right]$	$\left[94.8,95.2\right]$	$\left[94.9,95.3\right]$
	Sedentary	$\left[73.9,75.6\right]$	$\left[86.6,87.9\right]$	$\left[86.6,87.9\right]$	$\left[97.5,97.9\right]$	$\left[98.7,99.0\right]$	$\left[98.7,99.0\right]$
	Standing	$\left[59.5,61.9\right]$	$\left[79.8,81.8\right]$	$\left[79.8,81.8\right]$	$\left[93.6,94.1\right]$	$\left[97.1,97.5\right]$	$\left[97.1,97.5\right]$
Train Y	Walking	$\left[87.7,88.7\right]$	$\left[23.4,24.8\right]$	$\left[85.7,86.8\right]$	$\left[88.6,89.5\right]$	$\left[96.5,97.0\right]$	$\left[93.3,94.0\right]$
Test O *	Walking Upstairs	$\left[62.7,70.7\right]$	$\left[73.2,80.3\right]$	$\left[62.9,70.9\right]$	$\left[97.9,98.2\right]$	$\left[79.0,79.8\right]$	$\left[98.1,98.4\right]$
	Walking Downstairs	$\left[83.9,89.8\right]$	$\left[44.5,53.3\right]$	$\left[81.5,87.8\right]$	$\left[96.7,97.0\right]$	$\left[91.3,91.9\right]$	$\left[96.9,97.2\right]$
	Postural Transitions	$\left[74.4,79.5\right]$	$\left[78.8,83.6\right]$	$\left[86.5,90.4\right]$	$\left[96.9,97.3\right]$	$\left[96.0,96.4\right]$	$\left[96.6,97.0\right]$
	Sedentary	$\left[94.5,96.0\right]$	$\left[94.9,96.3\right]$	$\left[94.9,96.3\right]$	$\left[98.7,99.2\right]$	$\left[98.1,98.7\right]$	$\left[98.1,98.7\right]$
	Standing	$\left[92.8,94.7\right]$	$\left[88.5,90.8\right]$	$\left[88.5,90.8\right]$	$\left[93.7,94.7\right]$	$\left[96.4,97.2\right]$	$\left[96.4,97.2\right]$
Train O	Walking	$\left[61.1,64.5\right]$	$\left[21.5,24.4\right]$	$\left[62.5,65.8\right]$	$\left[97.6,98.3\right]$	$\left[97.6,98.3\right]$	$\left[97.3,98.0\right]$
Test Y *	Walking Upstairs	$\left[75.7,83.8\right]$	$\left[46.6,56.6\right]$	$\left[75.7,83.8\right]$	$\left[96.9,97.6\right]$	$\left[94.7,95.6\right]$	$\left[96.7,97.4\right]$
	Walking Downstairs	$\left[88.5,94.3\right]$	$\left[84.0,90.9\right]$	$\left[88.5,94.3\right]$	$\left[95.2,96.0\right]$	$\left[86.2,87.4\right]$	$\left[95.0,95.8\right]$
	Postural Transitions	$\left[45.0,58.3\right]$	$\left[73.9,84.7\right]$	$\left[82.6,91.6\right]$	$\left[96.2,97.0\right]$	$\left[92.8,93.8\right]$	$\left[95.4,96.2\right]$
	Sedentary	$\left[92.6,93.4\right]$	$\left[88.9,90.0\right]$	$\left[88.9,90.0\right]$	$\left[95.6,96.1\right]$	$\left[98.7,98.9\right]$	$\left[98.7,98.9\right]$
	Standing	$\left[72.2,74.0\right]$	$\left[82.6,84.1\right]$	$\left[82.6,84.1\right]$	$\left[97.2,97.5\right]$	$\left[97.2,97.6\right]$	$\left[97.2,97.6\right]$
Train Y&O	Walking	$\left[71.9,73.2\right]$	$\left[35.0,36.4\right]$	$\left[71.6,72.9\right]$	$\left[94.8,95.4\right]$	$\left[95.2,95.7\right]$	$\left[95.8,96.3\right]$
Test (Y&O) *	Walking Upstairs	$\left[69.3,75.1\right]$	$\left[48.3,54.8\right]$	$\left[69.3,75.1\right]$	$\left[96.3,96.7\right]$	$\left[87.4,88.0\right]$	$\left[96.4,96.7\right]$
	Walking Downstairs	$\left[89.6,93.3\right]$	$\left[65.1,71.4\right]$	$\left[89.7,93.4\right]$	$\left[93.6,94.0\right]$	$\left[89.2,89.8\right]$	$\left[93.7,94.1\right]$
	Postural Transitions	$\left[54.4,59.9\right]$	$\left[81.7,85.8\right]$	$\left[88.6,91.9\right]$	$\left[96.8,97.1\right]$	$\left[95.1,95.5\right]$	$\left[95.8,96.1\right]$

* The test data was obtained by virtually re-orienting the data from the younger (Y) and older (O) cohort as described in Section 3.1.

**Table A2:** Ninety-five percent confidence intervals for the sensitivity and specificity of each activity when hierarchical models for human activity recognition were developed with the best features.

	Activity	Sensitivity (%)			Specificity (%)		
	Train	100 Hz	100 Hz	40 Hz ${}^{†}$	100 Hz	100 Hz	40 Hz ${}^{†}$
	Test	100 Hz	40 Hz ${}^{†}$	100 Hz	100 Hz	40 Hz ${}^{†}$	100 Hz
	Sedentary	$\left[93.6,95.2\right]$	$\left[93.0,94.7\right]$	$\left[93.2,94.9\right]$	$\left[98.4,98.9\right]$	$\left[98.4,98.9\right]$	$\left[98.5,99.0\right]$
	Standing	$\left[88.8,91.1\right]$	$\left[88.6,90.9\right]$	$\left[87.8,90.2\right]$	$\left[96.1,96.9\right]$	$\left[95.7,96.5\right]$	$\left[96.3,97.1\right]$
Train Y	Walking	$\left[78.2,81.0\right]$	$\left[76.2,79.1\right]$	$\left[78.8,81.6\right]$	$\left[96.0,96.9\right]$	$\left[96.3,97.2\right]$	$\left[95.3,96.2\right]$
Test Y ${}^{⋆}$	Walking Upstairs	$\left[80.5,87.9\right]$	$\left[84.6,91.2\right]$	$\left[79.4,86.9\right]$	$\left[98.0,98.5\right]$	$\left[97.7,98.3\right]$	$\left[98.2,98.7\right]$
	Walking Downstairs	$\left[90.7,95.9\right]$	$\left[94.1,98.1\right]$	$\left[90.7,95.9\right]$	$\left[97.2,97.9\right]$	$\left[96.9,97.5\right]$	$\left[96.8,97.5\right]$
	Postural Transition	$\left[82.1,91.2\right]$	$\left[83.2,91.9\right]$	$\left[81.1,90.4\right]$	$\left[97.8,98.3\right]$	$\left[97.9,98.5\right]$	$\left[98.1,98.6\right]$
	Sedentary	$\left[87.5,88.8\right]$	$\left[87.7,88.9\right]$	$\left[87.2,88.5\right]$	$\left[98.6,98.9\right]$	$\left[98.5,98.8\right]$	$\left[98.6,98.9\right]$
	Standing	$\left[80.9,82.7\right]$	$\left[81.0,82.8\right]$	$\left[79.9,81.8\right]$	$\left[97.2,97.5\right]$	$\left[97.1,97.5\right]$	$\left[97.3,97.7\right]$
Train O	Walking	$\left[73.4,74.8\right]$	$\left[73.1,74.5\right]$	$\left[75.6,77.0\right]$	$\left[95.9,96.5\right]$	$\left[95.9,96.4\right]$	$\left[95.7,96.2\right]$
Test O ${}^{⋆}$	Walking Upstairs	$\left[54.2,62.5\right]$	$\left[58.8,67.0\right]$	$\left[49.3,57.7\right]$	$\left[96.4,96.8\right]$	$\left[96.4,96.8\right]$	$\left[97.3,97.6\right]$
	Walking Downstairs	$\left[83.9,89.8\right]$	$\left[85.8,91.4\right]$	$\left[82.8,88.9\right]$	$\left[94.0,94.5\right]$	$\left[93.7,94.2\right]$	$\left[94.2,94.7\right]$
	Postural Transition	$\left[89.9,93.3\right]$	$\left[87.6,91.3\right]$	$\left[90.3,93.6\right]$	$\left[94.9,95.3\right]$	$\left[95.3,95.7\right]$	$\left[94.4,94.9\right]$
	Sedentary	$\left[86.6,87.9\right]$	$\left[86.4,87.7\right]$	$\left[85.5,86.9\right]$	$\left[98.7,99.0\right]$	$\left[98.7,99.0\right]$	$\left[98.9,99.1\right]$
	Standing	$\left[79.8,81.8\right]$	$\left[80.1,82.0\right]$	$\left[77.1,79.1\right]$	$\left[97.1,97.5\right]$	$\left[97.1,97.4\right]$	$\left[97.5,97.8\right]$
Train Y	Walking	$\left[85.7,86.8\right]$	$\left[86.2,87.2\right]$	$\left[86.9,88.0\right]$	$\left[93.3,94.0\right]$	$\left[93.3,93.9\right]$	$\left[91.9,92.6\right]$
Test O ${}^{⋆}$	Walking Upstairs	$\left[62.9,70.9\right]$	$\left[69.0,76.5\right]$	$\left[62.6,70.5\right]$	$\left[98.1,98.4\right]$	$\left[98.2,98.5\right]$	$\left[98.5,98.7\right]$
	Walking Downstairs	$\left[81.5,87.8\right]$	$\left[83.6,89.6\right]$	$\left[84.1,90.0\right]$	$\left[96.9,97.2\right]$	$\left[96.9,97.3\right]$	$\left[96.5,96.9\right]$
	Postural Transition	$\left[86.5,90.4\right]$	$\left[86.0,90.0\right]$	$\left[86.5,90.4\right]$	$\left[96.6,97.0\right]$	$\left[96.8,97.2\right]$	$\left[96.9,97.3\right]$
	Sedentary	$\left[94.9,96.3\right]$	$\left[94.5,96.0\right]$	$\left[94.8,96.3\right]$	$\left[98.1,98.7\right]$	$\left[98.0,98.6\right]$	$\left[98.1,98.7\right]$
	Standing	$\left[88.5,90.8\right]$	$\left[88.1,90.4\right]$	$\left[88.4,90.7\right]$	$\left[96.4,97.2\right]$	$\left[96.0,96.9\right]$	$\left[96.4,97.2\right]$
Train O	Walking	$\left[62.5,65.8\right]$	$\left[59.4,62.8\right]$	$\left[64.3,67.6\right]$	$\left[97.3,98.0\right]$	$\left[97.5,98.2\right]$	$\left[97.2,97.9\right]$
Test Y ${}^{⋆}$	Walking Upstairs	$\left[75.7,83.8\right]$	$\left[80.3,87.6\right]$	$\left[72.9,81.3\right]$	$\left[96.7,97.4\right]$	$\left[96.3,97.0\right]$	$\left[97.6,98.2\right]$
	Walking Downstairs	$\left[88.5,94.3\right]$	$\left[90.2,95.5\right]$	$\left[86.5,92.8\right]$	$\left[95.0,95.8\right]$	$\left[94.5,95.3\right]$	$\left[95.2,96.0\right]$
	Postural Transition	$\left[82.6,91.6\right]$	$\left[83.2,91.9\right]$	$\left[83.2,91.9\right]$	$\left[95.4,96.2\right]$	$\left[95.6,96.3\right]$	$\left[94.8,95.6\right]$
	Sedentary	$\left[88.9,90.0\right]$	$\left[88.7,89.8\right]$	$\left[88.7,89.8\right]$	$\left[98.7,98.9\right]$	$\left[98.6,98.9\right]$	$\left[98.6,98.9\right]$
	Standing	$\left[82.6,84.1\right]$	$\left[82.5,84.0\right]$	$\left[80.5,82.1\right]$	$\left[97.2,97.6\right]$	$\left[97.1,97.4\right]$	$\left[97.6,97.9\right]$
Train Y&O	Walking	$\left[71.6,72.9\right]$	$\left[70.5,71.8\right]$	$\left[81.9,83.0\right]$	$\left[95.8,96.3\right]$	$\left[95.8,96.3\right]$	$\left[94.5,95.1\right]$
Test (Y&O) ${}^{⋆}$	Walking Upstairs	$\left[69.3,75.1\right]$	$\left[73.4,78.9\right]$	$\left[65.3,71.3\right]$	$\left[96.4,96.7\right]$	$\left[96.4,96.7\right]$	$\left[97.9,98.1\right]$
	Walking Downstairs	$\left[89.7,93.4\right]$	$\left[91.1,94.6\right]$	$\left[85.4,89.8\right]$	$\left[93.7,94.1\right]$	$\left[93.2,93.7\right]$	$\left[96.4,96.7\right]$
	Postural Transition	$\left[88.6,91.9\right]$	$\left[88.0,91.3\right]$	$\left[89.6,92.7\right]$	$\left[95.8,96.1\right]$	$\left[96.0,96.4\right]$	$\left[95.6,96.0\right]$

${}^{⋆}$ Test data were obtained by re-orienting the data from the younger (Y) and/or older (O) cohort (see Section 3.1); ${}^{†}$ IMU data were re-sampled at 40 Hz, barometer data were re-sampled at 20 Hz.
